# Microfossils, a Key to Unravel Cold-Water Carbonate Mound Evolution through Time: Evidence from the Eastern Alboran Sea

**DOI:** 10.1371/journal.pone.0140223

**Published:** 2015-10-08

**Authors:** Claudio Stalder, Agostina Vertino, Antonietta Rosso, Andres Rüggeberg, Claudius Pirkenseer, Jorge E. Spangenberg, Silvia Spezzaferri, Osvaldo Camozzi, Sacha Rappo, Irka Hajdas

**Affiliations:** 1 Department of Geosciences, University of Fribourg, Fribourg, Switzerland; 2 Department of Earth and Environmental Sciences, University of Milano-Bicocca, Milano, Italy; 3 Department of Biological, Geological and Environmental Sciences, University of Catania, Catania, Italy; 4 Institute of Earth Surface Dynamics, University of Lausanne, Lausanne, Switzerland; 5 Ion Beam Physics, Eidgenössische Technische Hochschule ETH Zürich, Zürich, Switzerland; Ben Gurion University of the Negev, ISRAEL

## Abstract

Cold-water coral (CWC) ecosystems occur worldwide and play a major role in the ocean's carbonate budget and atmospheric CO_2_ balance since the Danian (~65 m.y. ago). However their temporal and spatial evolution against climatic and oceanographic variability is still unclear. For the first time, we combine the main macrofaunal components of a sediment core from a CWC mound of the Melilla Mounds Field in the Eastern Alboran Sea with the associated microfauna and we highlight the importance of foraminifera and ostracods as indicators of CWC mound evolution in the paleorecord. Abundances of macrofauna along the core reveal alternating periods dominated by distinct CWC taxa (mostly *Lophelia pertusa*, *Madrepora oculata*) that correspond to major shifts in foraminiferal and ostracod assemblages. The period dominated by *M*. *oculata* coincides with a period characterized by increased export of refractory organic matter to the seafloor and rather unstable oceanographic conditions at the benthic boundary layer with periodically decreased water energy and oxygenation, variable bottom water temperature/density and increased sediment flow. The microfaunal and geochemical data strongly suggest that *M*. *oculata* and in particular Dendrophylliidae show a higher tolerance to environmental changes than *L*. *pertusa*. Finally, we show evidence for sustained CWC growth during the Alleröd-Younger-Dryas in the Eastern Alboran Sea and that this period corresponds to stable benthic conditions with cold/dense and well oxygenated bottom waters, high fluxes of labile organic matter and relatively strong bottom currents

## Introduction

Although cold-water corals (CWCs) are known since centuries, they became a major research "hot" topic only in the last two decades. Extensive studies (e.g., [1,2,3]) have helped to constrain their geographical distribution and their occurrence on the geological time scale but still little is known about the effects that environmental changes have on CWC mound development. Frame-building CWC species settle mainly on hard topographic highs (e.g., [1,3–6]), subvertical walls and overhangs [6–8] where a relatively strong hydrographic regime prevents corals from sediment smothering [9] and provide them with food (e.g., [10,11]). Recently, aquarium cultures of living CWC species collected from the North Atlantic and the Mediterranean Sea and their δ^13^C and δ^15^N values from coral tissues have revealed that CWCs are able to feed on a wide range of food sources including fresh macrozooplankton, fecal pellets, degraded phytodetritus, dissolved organic matter and bacteria (e.g., [11–14]).

Comprehensive studies (e.g., [1,15,16]) have demonstrated that the distribution of CWCs is largely driven by the chemo-physical properties of the surrounding water mass where, temperatures, salinities and dissolved oxygen contents usually range within 4–14°C, 31.7–38.8 and 2.6–7.2 ml l^-1^, respectively. Large-scale water masses characterizations in active CWC settings from the Celtic and Norwegian shelves and distributed over a wide bathymetric range (140–850 m water depth) have shown that living corals thrive within a water density gradient of sigma-theta (σ_θ_) = 27.35 to 27.65 kg m^-3^ [17]. In the Mediterranean Sea, living CWC colonies have been found in water densities of (σ_θ_) = 29.07 to 29.13 kg m^-3^ [7].

The interpretation of CWC paleorecords is usually difficult because of the large fluctuations in the sedimentation rates and the frequent hiatuses caused by strong bottom currents (e.g., [18,19]). Furthermore, in most cases CWC sediments consist entirely of biogenic fragments of different size and preservation that complicates the paleo-environmental interpretation of the sedimentary record and specific attribution to episodes of CWC growth, to temporary growth interruptions or to the demise of the CWC reef/mound in the past. Nevertheless, it is essential to understand the response of fossil CWC to climate and oceanographic changes to predict their future and to evaluate how much their existence will influence the total carbonate budget and the atmospheric CO_2_ on Earth [20].

During the last two decades, only few studies on live (stained) and dead (fossil) benthic foraminifera and ostracods associated to CWC ecosystems have been reported from the Norwegian shelf [21–25], the Porcupine Seabight and Rockall Trough [26–30], the Gulf of Cadiz and the Alboran Sea [31,32], the Ionian Sea [33], the Tuscan Archipelago [34] and Nova Scotia [35]. Several of those studies (e.g., [24,27,29]) allowed gaining further comprehension on the distribution of specific taxa according to sedimentary facies and microhabitats along CWC mounds and reefs. Freiwald and Schönfeld [22] showed evidence for predation of *Hyrrokin sarcophaga* on live CWC polyps whereas Margreth et al. [27] proposed the epibenthic species *Discanomalina coronata* as a potential bioindicator for living CWC reefs.

Compared to studies on microorganisms (foraminifera and ostracods), only few of numerous studies on live macro- and meiofauna from CWC settings focused on the fossil distribution (e.g., [36,33,37]). Both the skeletonised benthic micro and macrofauna studies associated to CWCs have clearly shown that foraminifera, ostracods and macrofauna may provide a powerful paleoproxy to understand lateral variability and evolution of CWC development through time (e.g., [25–27,33,36]).

We integrate studies on recent benthic macro- and microfauna from a CWC mound of the eastern Alboran Sea and their abundance during the last 13 ka, with special emphasis on scleractinians, bryozoans, foraminiferans and ostracods to relate their evolution through time and their response to paleoceanography modifications. For the first time we have cross-correlated biotic and geochemical proxies to interpret the evolution of a CWC mound.

### Geological and Oceanographic Settings

**Fig 1:**
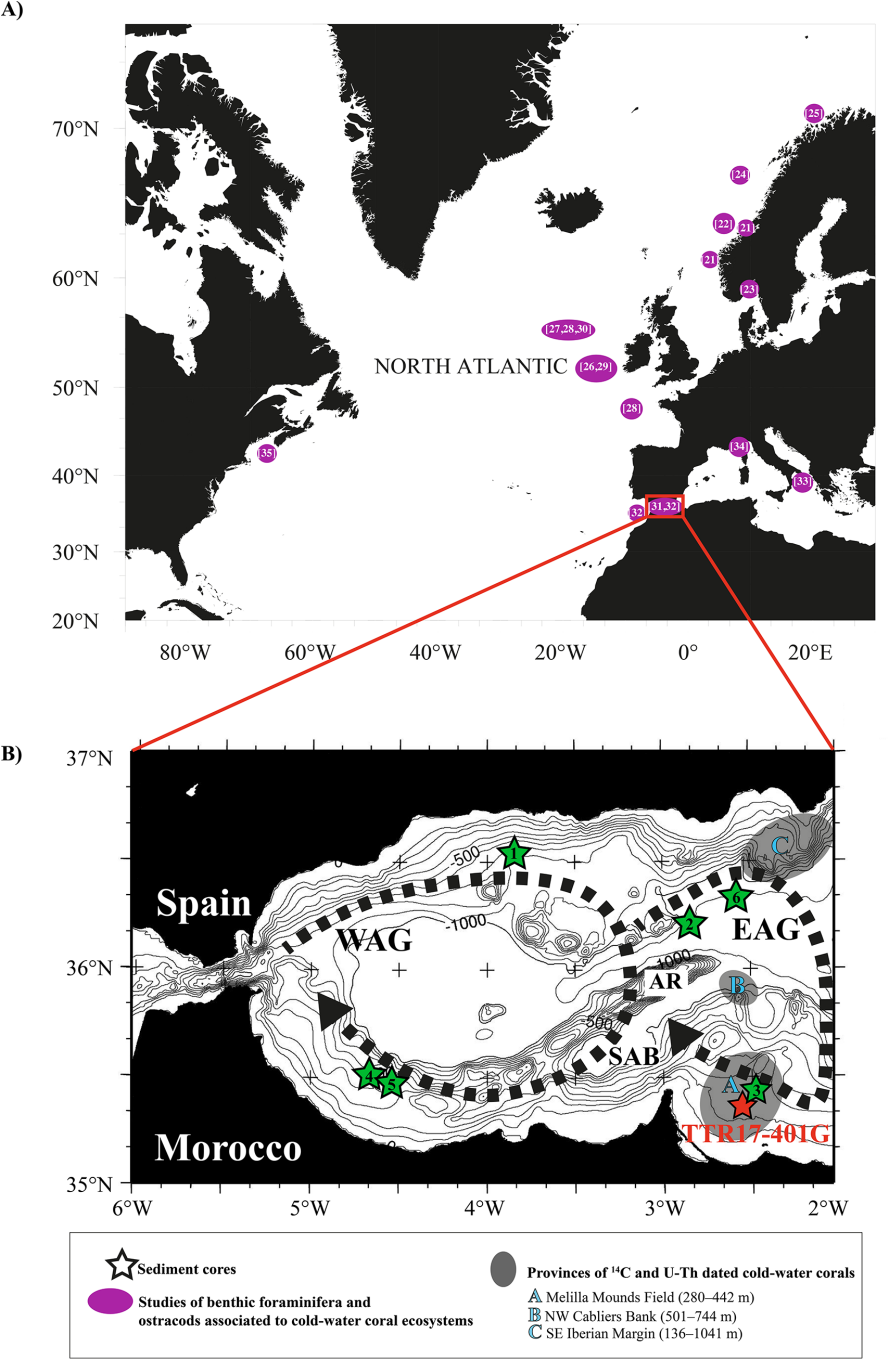
A. Map showing the major study areas of live (stained) and dead (fossil) benthic foraminifera and ostracods associated to cold-water coral ecosystems: the Norwegian shelf [21–25], the Porcupine Seabight and Rockall Trough [26–30], Nova Scotia [35], the Gulf of Cadiz and Alboran Sea [31, 32], the Ionian Sea [33] and the Tuscan Archipelago [34]. B. Bathymetric map of the Alboran Sea showing the surface-water circulation with the eastern (EAG) and western Alboran gyres (WAG), the Alboran Ridge (AR) and the South Alboran Basin (SAB). The red star shows the location of core TTR17-401G (251 m water depth) and the green stars the location and water depths of adjacent cores discussed in this study: 1, KS8230 (795 m); 2, TTR12-293G (1840 m); 3, GeoB13731-1 (362 m); 4, TTR17-MS419G (410 m); 5, TTR17-MS411G (370 m); 6, MD95-2043 (1841 m). The dashed areas indicate the location and water depth of cold-water coral samples from the Alboran Sea dated with ^14^C and U/Th (<20 ka BP): A, Melilla Mounds Field [42]; B, NW Cabliers Bank [43] and C, SE Iberian Margin [44]

The Alboran Sea in the western Mediterranean Sea is a ~400 km long and ~200 km wide basin with water depths not exceeding 2000 m (Fig 1) that exhibits a complex seafloor morphology with several sub-basins, ridges and seamounts [38]. Our study area is located in the South Alboran Basin (SAB), which is a NW-SE trending tectonically controlled basin bounding the southern flank of the Alboran Ridge [39]. Its formation started in the late Cretaceous as a consequence of crustal extension in a setting of overall convergence of the African and Eurasian plates [40]. This North-South convergence was reactivated in the latest Tortonian [41]. After post-Messinian times, active compressional structures such as the Alboran Ridge (Fig 1) or strike-slip faults such as the Nektor fault were produced [39].

The Melilla Mounds Field (MMF) is located in the southeastern Alboran Sea (western Mediterranean Sea) southeast to the Cape Tres Forcas (Fig 1). The submarine morphology of the MMF is characterized by carbonate mounds (Fig 1), which cover a surface of ~100 km^2^ within a water depth range of 250–600 m [45]. Similarly to the mounds in the north Atlantic, the mounds of the MMF form elongated and domed biogenic carbonate buildups with a diameter ranging from 48 m to 476 m, up to 100 m high above the seafloor, displaying a maximum length of 3000 m and mostly buried by a 1–12 m thick fine-grained sedimentary cover [42,45].

Radiocarbon dating suggests that the CWCs of the MMF started to develop during the late Pleistocene on unconformities and landslides [42]. Based on video survey, only a few living CWC colonies still occur in the MMF nowadays [46].

From an oceanographic point of view, the Alboran Sea basin is a peculiar basin strongly influenced by water exchange between the Atlantic Ocean and the Mediterranean Sea. Three main water masses characterize the modern water mass configuration in the Alboran Sea. The upper ~150–200 m of the water column are occupied by Modified Atlantic Water (MAW) (salinity = ~36.2 g/kg, T = ~15°C) flowing from the Atlantic Ocean through the Strait of Gibraltar towards the Algerian Basin [47,48]. The MAW is transformed in the eastern Mediterranean Sea between Rhodes and Cyprus into the Levantine Intermediate Water (LIW) [47,49]. It occurs in water depths of 200–600 m, with a salinity of ~38.4 g/kg and a mean temperature of ~13.3°C [47]. The Western Mediterranean Deep-Water (WMDW; salinity = ~38.4 g/kg, T = ~12.8°C), formed in the Gulf of Lions (SE France) flows below the LIW in the deepest part of the Alboran Basin [47,50]. The WMDW flows towards the Atlantic Ocean and is topographically forced to shoal at ~300 m water depth when passing the sill of Gibraltar [51]. The Mediterranean Outflow Water (MOW), which flows into the Atlantic Ocean along the Iberian margin, is composed of LIW and WMDW [52].

In the Alboran Sea, the inflowing MAW forms two anticyclonic gyres of ~100 km in diameter: the Western Alboran Gyre (WAG) and the Eastern Alboran Gyre (EAG) (Fig 1) [53]. The two gyres are roughly situated over the western and eastern Alboran basins with respective maximum depths of 1200 and 1800 m. Both are separated from each other by the Alboran Ridge [54]. The WAG and EAG do not have very stable positions or behaviours given the strong seasonal variations in the surface circulation of the SAB [55]. In summer, both gyres are rather constant, but during winter the WAG often migrates eastwards and the EAG even disappears due to higher MAW inflow and MOW outflow. Furthermore, stronger westerly winds [56] develop a jet along the African coast instead of the gyre [54].

The modern Alboran Sea is generally oligotrophic with the exception of two areas of high primary productivity [57]. The first area is situated on the northern limb of the WAG, where westerly winds cause the upwelling of nutrient-rich subsurface waters and lead to production rates of up to 200 g C m^-2^ yr^-1^ [58,59]. The second elevated primary productivity centre is located along the Almeria-Oran Front and is triggered by the density contrast between MAW and resident Mediterranean surface water with increased salinity [57,60].

## Material and Methods

The 560 cm long sediment core 401G was recovered in the Melilla Mounds Field (MMF) at a water depth of 251 m (35°19.273'N, 02°34.001'W) (Fig 1) during the Training-Through-Research TTR17 cruise in 2008 [61]. It consists of alternating layers of clayey to sandy mud bearing CWC fragments up to 10 cm long and other benthic macrofaunal components. The gravity core was sampled each 20 cm for geochemical and micropaleontological investigations. Samples were processed following standard procedures for foraminiferal preparation (see [62,63]). Approximately 10 g of dry bulk sediment per sample was washed through three mesh sieves (63, 125 and 250 μm) and at least 200 specimens per fraction were counted and glued on plummer-cells for archive. If the residue contained more than the target number of 300 benthic foraminifera in a single fraction, its volume was split with a dry splitter. If the residue contained less than 300 specimens, all specimens were counted. We decided to focus on specimens larger than 125 μm (S1 Table) to exclude smaller forms, which are often displaced by redeposition [64], and to make the data comparable to other benthic foraminiferal studies in adjacent areas (e.g., [65–68]). The planktonic foraminifera and ostracods were identified on the fraction > 250 μm following similar procedures (S2 and S3 Tables). The planktonic to benthic (P/B) ratio has been calculated based on the > 250 μm size fraction to avoid overestimation of the ratio due to the redeposition of smaller specimens.

Quantitative analyses of benthic foraminifera were performed with the Software PRIMER6 [69]. The dataset was double-square root transformed to limit the contribution of most abundant and ubiquitous species [70] and the Bray-Curtis (dis)Similarity Term Analysis was calculated [71]. The same similarity matrix used for Bray-Curtis (dis) similarities was used also to obtain the non-metric Multi Dimensional Scaling (nMDS) plot [72].

All recognizable entire specimens and skeletal fragments larger than 1 mm were counted and identified to the lowest possible taxonomic level (family to species, with exception of Asterozoa and Decapoda identified at subphylum and order level, respectively) (S4–S6 Tables). Moreover, due to the small size of important bryozoan species belonging to Candidae and Crisia, observations on presence/absence of bryozoan taxa were performed also on the 0.5–1 mm sediment grain fraction.

**Fig 2:**
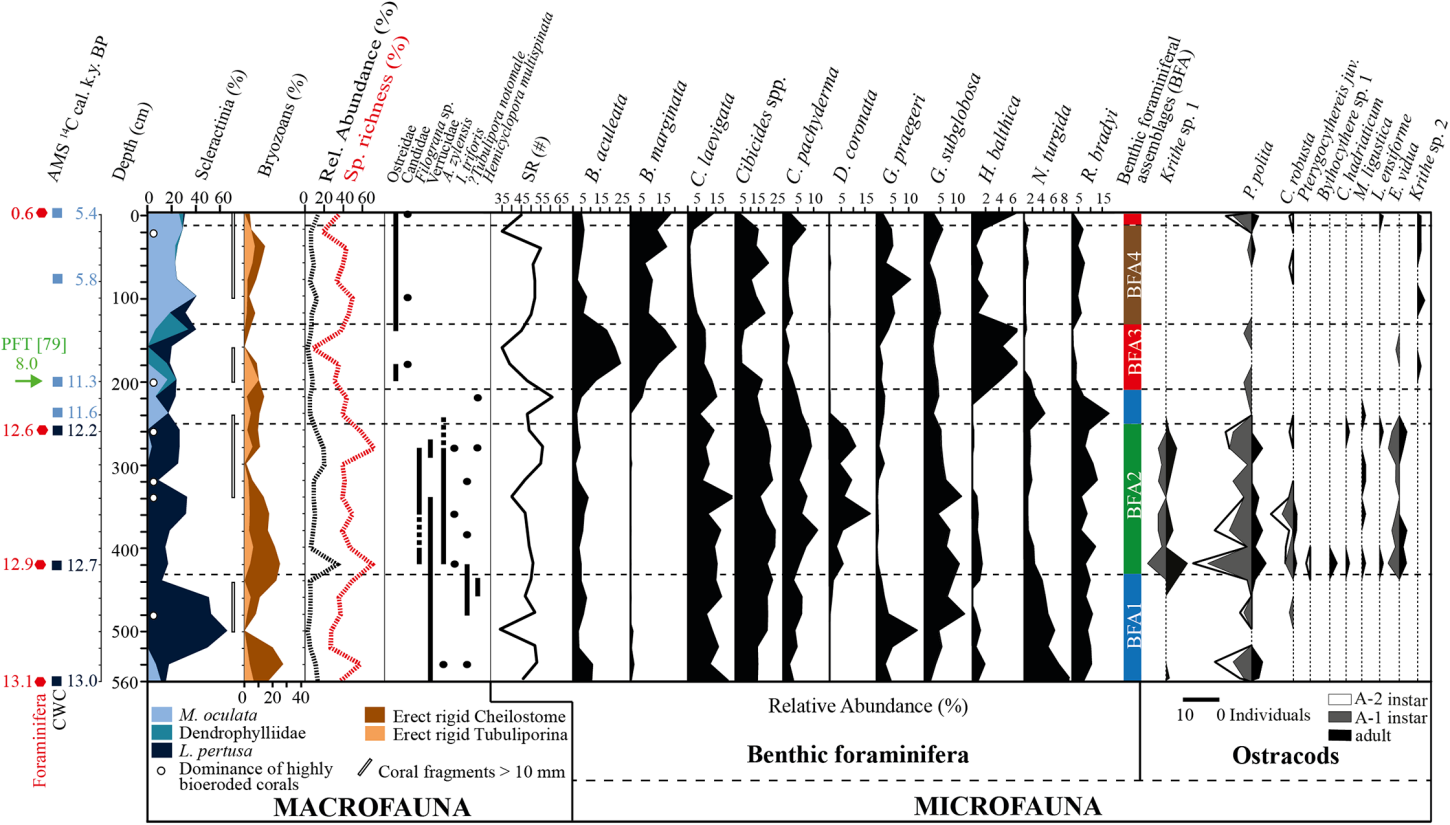
Distribution of main macrofaunal components, benthic foraminifera, benthic foraminifera assemblages (BFA) and ostracods in core TTR17-401G. The chronology of the core is based on AMS ^14^C ages of foraminifera and corals and the planktonic foraminiferal turnover (PFT), expressed as a maximum age. The relative abundance of corals and bryozoans is expressed as percentage of the total number of counted macrofauna specimens per sample. The relative abundance of all macrofaunal specimens per sample (black dotted line) is expressed as percentage of the total number of counted specimens in the core. The relative taxonomic richness per sample (red dotted line) is expressed as percentage of the total number of macrofauna taxa found in the core and does not include scleractinian taxa. Benthic foraminiferal species richness (SR) is expressed as the total number of species found in each sample. The dashed lines display changes in the benthic foraminiferal assemblages.

In order to outline the main results of the macrofauna analysis in Fig 2 the relative abundance of the two dominant taxonomic groups (Scleractinia, Bryozoa) collected in the sediment fraction 1–10 mm are presented. Two main subgroups (“erect rigid Cheilostome” and “erect rigid Tubuliporina”, Fig 2, S5 Table) were selected among bryozoans. They include the most representative species in terms of abundance and abundance variation along the core.

Radiocarbon dating was performed at the Eidgenössische Technische Hochschule (ETH) Zürich using the accelerator mass spectrometry (AMS) technique. From selected samples, benthic foraminifera were picked until at least 5–10 mg of pure carbonate were obtained. The species *Discanomalina coronata* lives attached to a hard substrate and is associated to the CWC ecosystem [27] and was chosen wherever possible. Alternatively, the epibenthic foraminifera *Cibicides lobatulus* was picked. Specimens were cleaned in ultrasounds to remove eventual contamination. Coral fragments (25–50 mg) used for radiocarbon dating were selected according to their preservation and further treated by standard chemical leaching procedures. Table 1 summarizes the scleractinian species used for the AMS ^14^C dating. They were dissolved in concentrated phosphoric acid [73] and the extracted carbon dioxide was converted into graphite as described by [74]. All ages are corrected for ^13^C and, assuming a reservoir age correction of 400 years, the ^14^C ages were converted to calendar years (cal. yr BP; P = AD 1950) using the Marine13 calibration curve [75] and software OxCalV4.2.4 [76]. All ages are reported as kiloyears before present (ka BP; Table 1).

**Table 1:** Radiocarbon ^14^C ages of sediment (benthic foraminifera) and cold-water corals. All ages are corrected for a reservoir age of 400 years.

Core	Core depth	Material	Sample ID ♯	14C-age	1σ error	2σ range cal. age	Median probability age
	(cm)			(years)	(±years)	(years BP, P = AD 1950)	(years BP)
TTR17–401G	0	Fo–Lobatula	ETH-55620	1055	28	552–671	611
TTR17–401G	0	CWC–Mo	ETH-55621	5073	30	5316–5536	5426
TTR17–401G	80	CWC–Mo	ETH-57100	5466	28	5746–5912	5829
TTR17–401G	200	CWC–Mo	ETH-57101	10302	35	11168–11483	11326
TTR17–401G	240	CWC–Mo	ETH-57102	10476	35	11362–11862	11612
TTR17–401G	260	Fo–Coronata	ETH-55622	11146	56	12558–12763	12660
TTR17–401G	260	CWC–Lo	ETH-55623	10770	39	12036–12448	12242
TTR17–401G	420	Fo–Coronata	ETH-55624	11432	57	12731–13076	12903
TTR17–401G	420	CWC–Lo	ETH-55625	11231	39	12610–12824	12717
TTR17–401G	560	Fo–Lobatula	ETH-55626	11675	65	12987–13316	13151
TTR17–401G	560	CWC–Lo	ETH-55627	11553	40	12880–13166	13023

Fo = Foraminifera; CWC = Cold-water corals, Mo = Madrepora oculata, Lo = Lophelia pertusa

The stable isotope analyses were performed at the Stable Isotopes Laboratory of the University of Lausanne. Carbon and oxygen stable isotope composition of benthic (*Cibicides lobatulus*) and planktonic (*Globigerina bulloides*) foraminifera (Table 2) were determined with the Thermo Fisher Scientific carbonate preparation device and GasBench II connected to a Delta Plus XL isotope ratio mass spectrometer (IRMS). Between 5 and 15 specimens of each species were picked in the > 250 μm and cleaned twice in an ultrasonic bath. The stable carbon and oxygen isotopic ratios are reported in delta (δ) notation as per mil (‰) deviation relative to Vienna Pee Dee Belemnite (VPDB) standard. The standardization of the δ^13^C and δ^18^O values relative to the VPDB scale was done by calibration of the reference gas and working standards with IAEA standards. Analytical uncertainty (1 σ), monitored by replicate analyses of the international calcite standard NBS-19 and the laboratory standards Carrara Marble was not greater than ±0.05‰ for δ^13^C and ±0.1‰ for δ^18^O. Stable carbon isotope composition of the organic carbon (δ^13^C_org_) was determined by flash combustion on a Carlo Erba 1108 elemental analyzer (EA) connected to a Thermo Fisher Scientific Delta V IRMS that was operated in the continuous helium flow mode via a Conflo III split interface (Table 2). The reproducibility of the EA-IRMS measurement is better than ±0.1%. The accuracy of analyses was assessed using international reference standards.

**Table 2:** Geochemical data of core TTR17-401G. Are shown total organic carbon (TOC), mineral carbon (MINC), hydrogen index (HI), oxygen index (OI), planktonic (*Globigerina bulloides*) and benthic (*Cibicides lobatulus*) δ^18^O and δ^13^C, δ^13^C_org_ and grain-size distribution.

	Rock-Eval pyrolysis				Stable isotopes (‰ VPDB)					Grain-size (%)			
Depth (cm)	TOC [%wt.]	MINC [%wt.]	HI [mg HC/g TOC]	OI [mg CO_2_/g TOC]	δ^13^C_org_	δ^13^C_lobatula_	δ^18^O_lobatula_	δ^13^C_bulloides_	δ^18^O_bulloides_	>250 μm	250–125 μm	125–63 μm	<63 μm
0	0.7	5.76	99	278	-22.2	0.1	1.3	-0.8	0.6	58.29	1.11	0.68	39.91
20	1.07	3.71	102	212	-21.8	0.9	1.4	-1.1	0.8	15.61	0.33	0.92	83.14
40	0.98	3.8	84	211	-22.2	0.4	0.9	-0.5	0.8	14.05	0.83	1.83	83.3
60	0.86	5.34	90	224	-21.7	0.4	1.1	-1.2	0.6	50.58	0.41	1.49	47.52
80	1.03	3.74	95	224	-21.7	0.9	1.1	-0.6	0.9	14.55	0.61	2.42	82.41
100	1.14	3.95	90	191	-21.4	0.2	1.1	-0.5	0.9	31.77	0.87	0.77	66.59
120	1.05	3.73	98	221	-21.6	0.9	1.6	-0.7	1	14.52	0.98	1.09	83.41
140	0.93	4.52	92	213	-22	1	1.6	-0.5	0.9	45.58	0.92	0.5	53
160	0.98	3.23	82	220	-21.9	0.1	1.6	-1.1	0.7	1.28	0.46	0.74	97.52
180	0.97	3.43	86	219	-21.9	0.6	1.5	-1.1	0.6	41.42	0.79	1.8	55.99
200	0.87	5.11	78	202	-21.9	0.3	1.2	-1.4	0.4	38.87	0.92	3.72	56.5
220	0.95	4.35	102	195	-21.3	0.4	2.5	1.3	1.5	5.92	1.43	2.89	89.76
240	0.98	4.26	119	180	-21.1	1.2	2.5	0.6	2.3	42.82	0.73	5.25	51.19
260	0.71	7.03	112	243	-20.7	0.9	2.5	0.1	2.5	67.92	0.92	3.36	27.8
280	0.87	5.97	110	183	-20.8	0.9	2.7	0	2.2	54.34	1.28	2.01	42.37
300	0.8	6.7	105	220	-21.1	0.9	2.7	0.3	2.5	53.58	1.07	2.39	42.96
320	1.11	4.79	131	168	-21.2	1.1	2.9	-0.6	2.3	25.25	1.04	3.47	70.24
340	0.82	5.5	136	225	-21.3	0.8	2.6	0.4	2.5	48.8	1.19	2.59	47.41
360	0.92	5.51	119	186	-21.1	1	2.7	-0.7	1.7	25.66	2.96	5.36	66.02
380	0.85	4.83	124	187	-20.8	0.8	2.7	-0.5	2.6	12.53	2.93	10.03	74.51
400	0.99	4.71	112	179	-21.2	1	2.9	0.1	2.4	6.96	2.87	8.48	81.69
420	0.58	6.3	96	239	-21	0.8	2.6	-0.6	1.3	23.69	5.78	12.13	58.4
440	0.88	4.43	111	178	-21.4	0.9	2.8	-0.7	2.2	8.85	0.82	3.68	86.65
460	0.99	4.3	115	182	-21	1	2.7	0.5	2.6	15.49	1.18	2.65	80.67
480	0.96	4.09	119	177	-21	1.1	2.7	0.2	2.6	6.59	1.39	2.85	89.18
500	1.06	4.28	132	171	-21.2	0.7	2.8	-0.8	2.4	8.51	0.5	2.36	88.63
520	1	4.24	159	186	-21.2	0.2	2.6	-1	2.3	1.8	0.68	2.48	95.04
540	0.94	4.69	127	164	-21.5	-0.3	2.5	-1.1	1.8	2.7	0.84	2.95	93.51
560	0.68	5.79	117	199	-21	0.7	2.4	-1.1	1.9	4.97	1.31	5.5	88.23

Total organic carbon (TOC) content (in weight %) was determined at the laboratory of Sediment Geochemistry at the University of Lausanne on about 100 mg bulk sediment using the Rock-Eval6 technology and following the standard rock pyrolysis [77,78]. The Hydrogen Index (HI), expressed in mg HC/g TOC, displays the total amount of pyrolyzed hydrocarbons resulting from the cracking of non-volatile organic matter (HI = S_2_x100/TOC) and the Oxygen Index (OI, in mg CO_2_/g TOC) which accounts for the amount of CO_2_ generated during the pyrolysis of the kerogen (OI = S_3_x100/TOC), both normalized to TOC. Additional parameter provided by the Rock-Eval6 is the Mineral Carbon (MINC), which represents the percentage of carbon derived from inorganic sources. All Rock-Eval data are given in Table 2.

## Results

### Chronology

The chronology of the cores is constraint by 7 AMS ^14^C dating on CWC fragments and 4 on benthic foraminifera coupled to the distribution of planktonic foraminifera (Fig 2, Table 1). Discrepancies between coral and benthic foraminifera ages are a common feature in CWC mounds (e.g., [19, 26, 42]). Coral ages indicate the times when the organisms lived while ages from benthic foraminifera represent the sedimentation history of the CWC mound. However, both can be used for paleoceanographic comparisons but interpretations should be related to the organism. The radiocarbon dating reveals that core TTR17-401G covers the time span 0.6–13.1 ka BP, thus reaching back to the transition from the Alleröd interstadial to the Younger-Dryas (YD) cold event (12.9–11.5 ka BP). The calculated linear sedimentation rates (LSR) indicate that the sedimentation was shifting between extreme values of 611 cm/ka from the base to 260 cm, and 20.55 cm/ka from 260 cm to the top of the core.

The dated CWC fragments yield ages ranging from 5.4 at the top of the core to 13 ka at its base (Fig 2). Apparently, the CWCs stopped growing 5.4 ka at the core top, which has a sediment age of 0.6 ka. This indicates the presence of a hiatus close to the core top with possible erosion of sediments. Because of the age differences between corals and sediments, the sediment record of core TTR17-401G will therefore be expressed in all figures versus core depth and not versus age.

**Fig 3:**
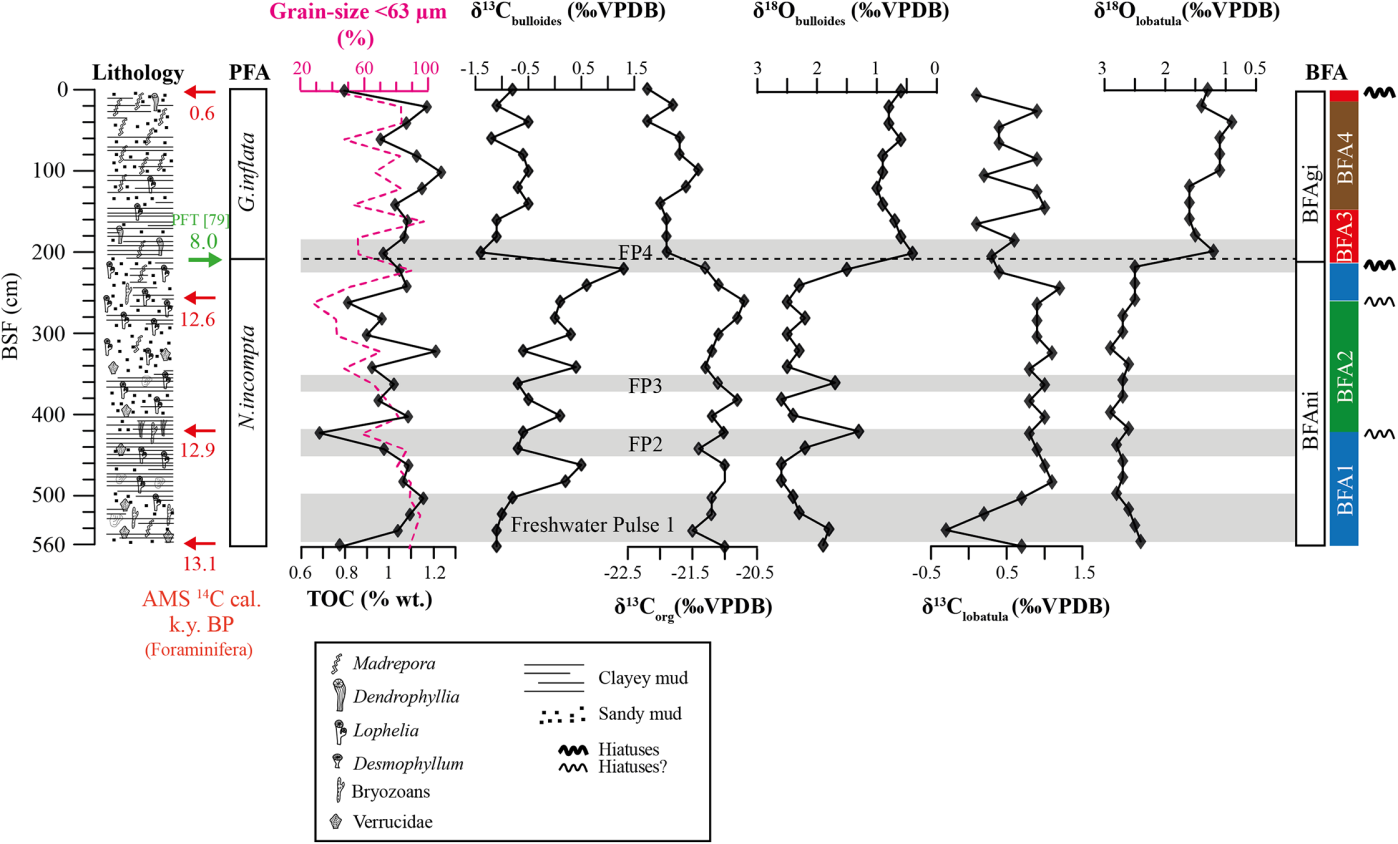
Multi-proxy record from core TTR17-401G. Are displayed the lithology with main macrofaunal components, radiocarbon ages of sediment (foraminifera), grain-size distribution (<63 μm), total organic carbon (TOC), δ^13^C_org_, δ^13^C and δ^18^O of benthic and planktonic foraminifera. Benthic foraminiferal assemblages (BFA) are shown according to the level of the Bray-Curties Similarity: BFAni and BFAgi (39%) and BFA1-BFA4 (54%). Dashed line indicates the turnover in the planktonic foraminiferal assemblage (PFA) at ca. 8 ka BP [79]. Freshwater pulses 1–4 correspond to possible freshening events of the (sub-) surface waters.

The distribution of planktonic foraminifera in the core shows the occurrence of two major intervals, the first lasting from 560 to 200 cm and dominated by *Neogloboquadrina incompta* and the second from 200 cm to the top and dominated by *Globorotalia inflata* (Figs 2 and 3). This planktonic foraminiferal turnover (PFT) has been well described in the Alboran Sea by Rohling et al. [79] and assumed to have occurred around 8 ka BP. Around this core depth another hiatus occurred as indicated by the different ages between sediment (8 ka) and corals (~11 ka) (Fig 2).

### Stable carbon and oxygen isotopes in foraminifera

The planktonic δ^18^O values decrease towards the top of the core and range between 0.4 and 2.6‰ (Fig 3, Table 2). In the *N*. *incompta* dominated interval, the planktonic δ^18^O values (1.3–2.6‰) are higher than during the *G*. *inflata* interval (0.4–1‰). In the interval dominated by *N*. *incompta*, the planktonic δ^18^O values vary between 2.2 and 2.6‰, except for samples 540–560, 420, 360 and 220 cm showing relatively large negative excursions of 1.9, 1.8, 1.3, 1.7 and 1.5‰ respectively (Fig 3, Table 2).

The benthic δ^18^O curve shows a similar pattern as the planktonic δ^18^O curve with higher values of 2.4–2.9‰ in the interval dominated by *N*. *incompta* compared to lower values in the *G*. *inflata* interval (0.9–1.6‰) (Fig 3, Table 2). Highest values of 2.9‰ are reached during the early YD (12.6–12.9 ka BP). In the *G*. *inflata* interval benthic δ^18^O values show a decrease in two steps, one from 120–200 cm (1.2–1.6‰) and another one from 40–100 cm (0.9–1.1‰) followed by an increase in the last 20 cm (1.3–1.4‰) (Fig 3). The relatively large negative excursions observed in the planktonic δ^18^O values coincide with decreases in the benthic δ^18^O, from which the most prominent occurs at the transition from the interval dominated by *N*. *incompta* to *G*. *inflata* with a decrease of -1.1‰ in the planktonic and -1.3‰ in the benthic δ^18^O.

The planktonic and benthic δ^13^C display values ranging from -1.4 to 1.3‰ and -0.3 to 1.2‰ respectively and show roughly a similar evolution throughout the core. The highest planktonic δ^13^C value was measured in the interval 220–480 cm where the values are generally of a magnitude higher than for the lower and upper samples (Fig 3). At 200 cm, the planktonic δ^13^C decrease of -2.7‰ coincides with the transition from the *N*. *incompta* to the *G*. *inflata* interval (Fig 3). Similarly, higher benthic δ^13^C were measured from 480 cm to 240 cm, where values drop from 1.2 to 0.4‰, thus slightly before the planktonic δ^13^C (Fig 3). The interval 240–480 cm is characterized by extremely stable benthic δ^13^C signal compared to measured in the lower and upper parts of the core.

### Sediment characterization: Rock-Eval pyrolysis and stable carbon isotopes of TOC

The Total Organic Carbon shows values ranging from 0.58–1.14% (Fig 3, Table 2). TOC values tend to decrease from the base to 260 cm with three marked minima at 560, 420 and 260 cm (Fig 3). TOC values increase from 240 cm to a maximum of 1.14% at 100 cm before decreasing again in the uppermost part of the core (Fig 3). The TOC content displays a positive correlation to the mud fraction (Fig 3).

**Fig 4:**
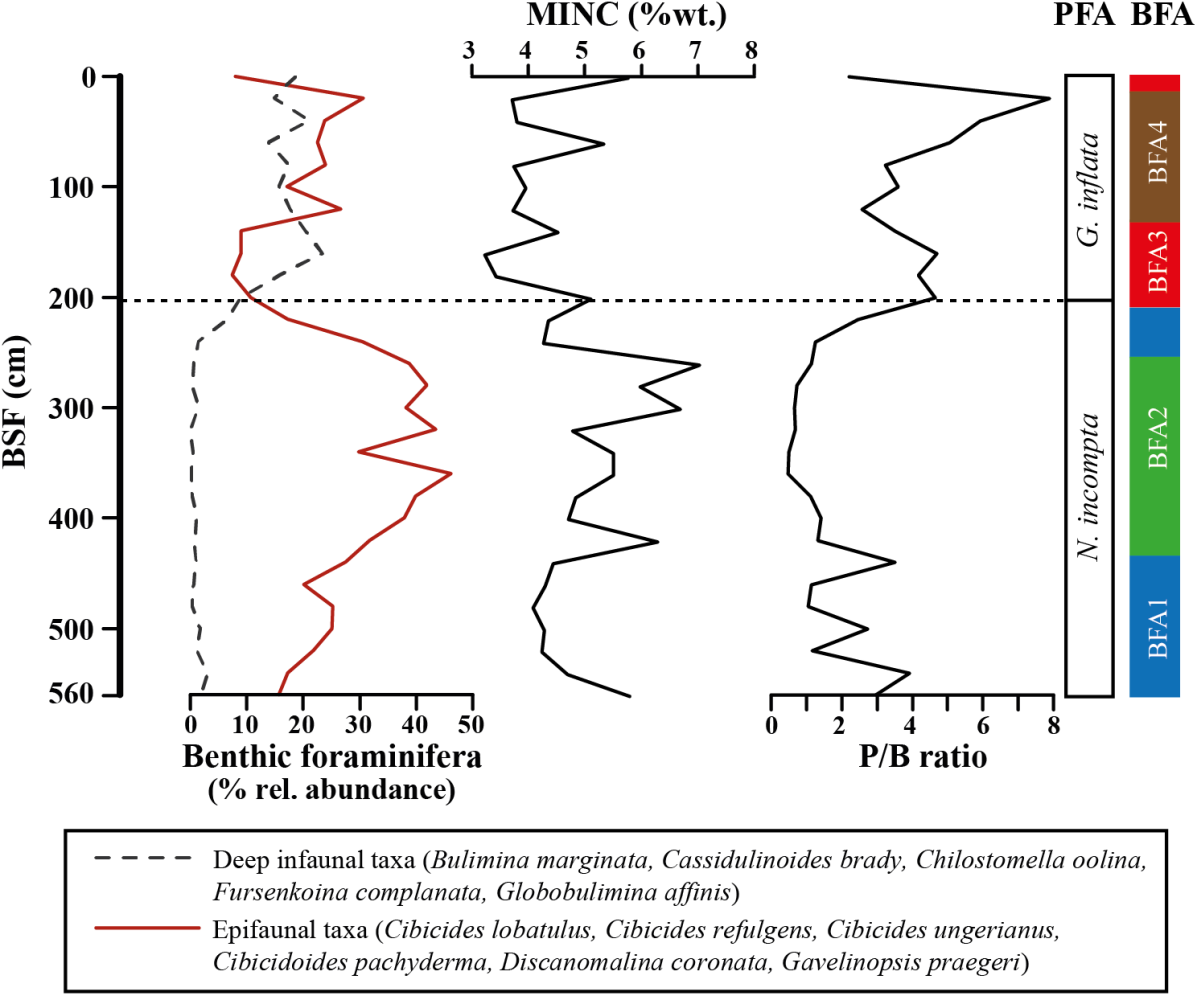
Relative abundances of selected epibenthic and infaunal benthic foraminifera. Mineral carbon (MINC) content and P/B ratio are plotted against the benthic foraminiferal assemblages (BFA). The dashed line indicates the planktonic foraminiferal turnover [79].

The Mineral Carbon content is higher (4.71–7.03%) in the interval 260–420 cm compared to the intervals 440–560 cm (4.09–5.79%) and 0–240 cm (3.23–5.76%). The MINC shows a relatively strong negative correlation to the P/B ratio (Fig 4).

**Fig 5:**
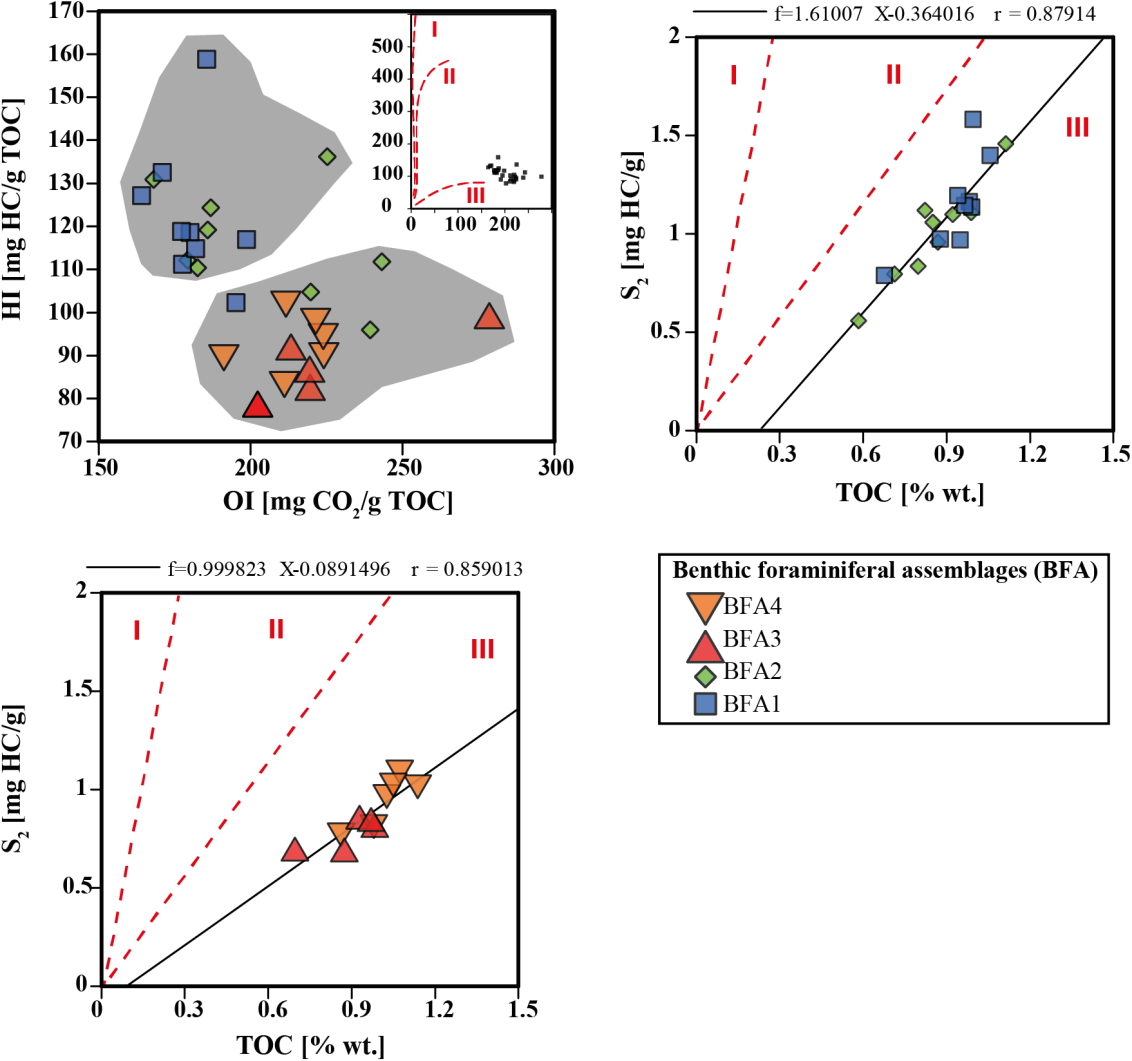
Pseudo-Van Krevelen HI versus OI plots showing the distribution of samples from core TTR17-401G and S2 versus Total organic carbon (TOC) diagrams for samples BFA1-BFA2 and BFA3-BFA4. Regression curves are also shown for each diagram. Boundaries between the kerogen type fields are from Langford and Blanc-Valleron [80].

The Hydrogen and Oxygen Index vary from 78–158 mg HC/g TOC and 164–278 mg CO_2_/g TOC respectively (Fig 5; Table 2). A relatively clear trend can be recognized with higher HI and lower OI in the interval dominated by *N*. *incompta* and lower HI and higher OI in the interval dominated by *G*. *inflata* (Fig 5; Table 2). The values of δ^13^C_org_ range from -22.2‰ (0 cm) to -20.7‰ (260 cm). The δ^13^C_org_ follows a similar trend as the HI with higher values (-21.5–-20.7‰) in the *N*. *incompta* interval and lower values (-22.2–-21.4‰) in the *G*. *inflata* interval (Fig 3, Table 2). Highest δ^13^C_org_ values are measured within interval 260–420 cm (Fig 3). The δ^13^C_org_ signal follows well the planktonic δ^18^O and δ^13^C signal with marked minima at 540, 440, 340 and 200 cm.

### Micropaleontology

Core TTR17-401G is characterized by a conspicuous distribution of the large (>250 μm) planktonic and benthic foraminifera (S1 and S2 Tables). The target value of 200 benthic specimens per fraction could be reached only in the samples from 260–420 cm where most of the samples were split. In all other samples, all specimens of the residue were counted and a minimum was obtained at 500 cm (8 specimens) and a maximum at 540 cm (110 specimens). The planktonic foraminifera showed an opposite trend with lowest scores (71–202 specimens) between 220 and 520 cm and the target value reached only at 380 cm (S2 Table). The planktonic foraminifera contribution to the total foraminifera fauna is well documented in the P/B ratio (Fig 4).

### Benthic foraminifera–univariate distribution

In total, 138 benthic foraminifera species (unstained) belonging to 84 genera have been identified in core TTR17-401G (S1 Table). Species richness (SR) varies from 34 at 500 cm to 61 at 220 cm. The most common species found in this core are *Bulimina aculeata*, *Bulimina marginata*, *Cassidulina laevigata*, *C*. *lobatulus*, *Cibicides refulgens*, *Cibicides ungerianus* (grouped as *Cibicides* spp. in Fig 2), *D*. *coronata*, *Gavelinopsis praegeri*, *Globocassidulina subglobosa*, *Hyalinea balthica*, *Nonionella turgida* and *Rosalina bradyi* (Fig 2). All these species, except *D*. *coronata*, have also been reported from recent and sub-recent sediments from shallower water depths in the Alboran Sea [68]. This latter species was however reported from a similar environment in the western Alboran Sea [31] (Fig 1). *D*. *coronata* has been widely documented on CWC ecosystems from the Norwegian shelf (e.g., [24,25]) and Porcupine Seabight and Rockall Trough [27,29].

Among these common species, *B*. *aculeata*, *B*. *marginata* and *C*. *laevigata* belong to the most abundant species accounting up to 25%, 20% and 22% of the total benthic foraminifera fauna respectively. The infaunal *B*. *aculeata* is frequent through the entire core with decreasing abundances from the base to 240 cm followed by elevated abundances (12–25%) between 140 and 200 cm before decreasing again towards the top of the core (Fig 2). With only very few abundances in the interval 240–560 cm (0–1.6%), the distribution of *B*. *marginata* is mostly restricted to the upper part of the core where it becomes a dominant species. A strong contribution of *B*. *marginata* occurs in the interval 140–200 cm (7–20%) together with elevated abundances of *B*. *aculeata* and in the uppermost 40 cm (12–18%). *Cassidulina laevigata* is frequent in all samples but is clearly decreasing from 220 cm to 20 with lowest scores at 40 cm (1.3%) (Fig 2). Other important taxa are the epibenthic species *C*. *lobatulus*, *C*. *refulgens*, *C*. *ungerianus* and *C*. *pachyderma* (Fig 2). They all occur throughout the core but show highest abundances in the interval 260–420 cm with 16–26% for *Cibicides* spp. and 5.3–11% for *C*. *pachyderma* and lowest abundances between 140 and 180 cm with 4.1–5.8% and 0.9–2.2% respectively (Fig 2). A similar distribution pattern can be recognized for the epibenthic *R*. *bradyi* (Fig 2). A particular distribution can be observed for the relatively large epibenthic *D*. *coronata*, which occurs significantly (6–17%) only in the interval 260–420 cm (Fig 2). The epibenthic *G*. *praegeri* is relatively abundant only in the intervals 440–560 cm (1.8–13%) and 20–120 cm (3.2–11%). The opportunistic epibenthic *H*. *balthica* is occurring in very low abundances in the interval 220–560 cm and is completely missing in samples 460, 300–320 and 240 cm. This species shows highest abundances in the interval 140–200 cm (4.2–6.9%) and at the top of the core (6.9%).

The relatively small opportunistic species *N*. *turgida* becomes successively less abundant towards the top of the core and shows highest contribution to the total fauna between 420–560 cm (up to 9%) and is completely absent in samples 160–180 cm and 0 cm (Fig 2). The infaunal *G*. *subglobosa* is frequent in the entire core but is clearly more abundant in the interval 260–520 cm accounting for up to 13% (Fig 2).

#### Benthic foraminiferal assemblages–multivariate distribution

**Fig 6:**
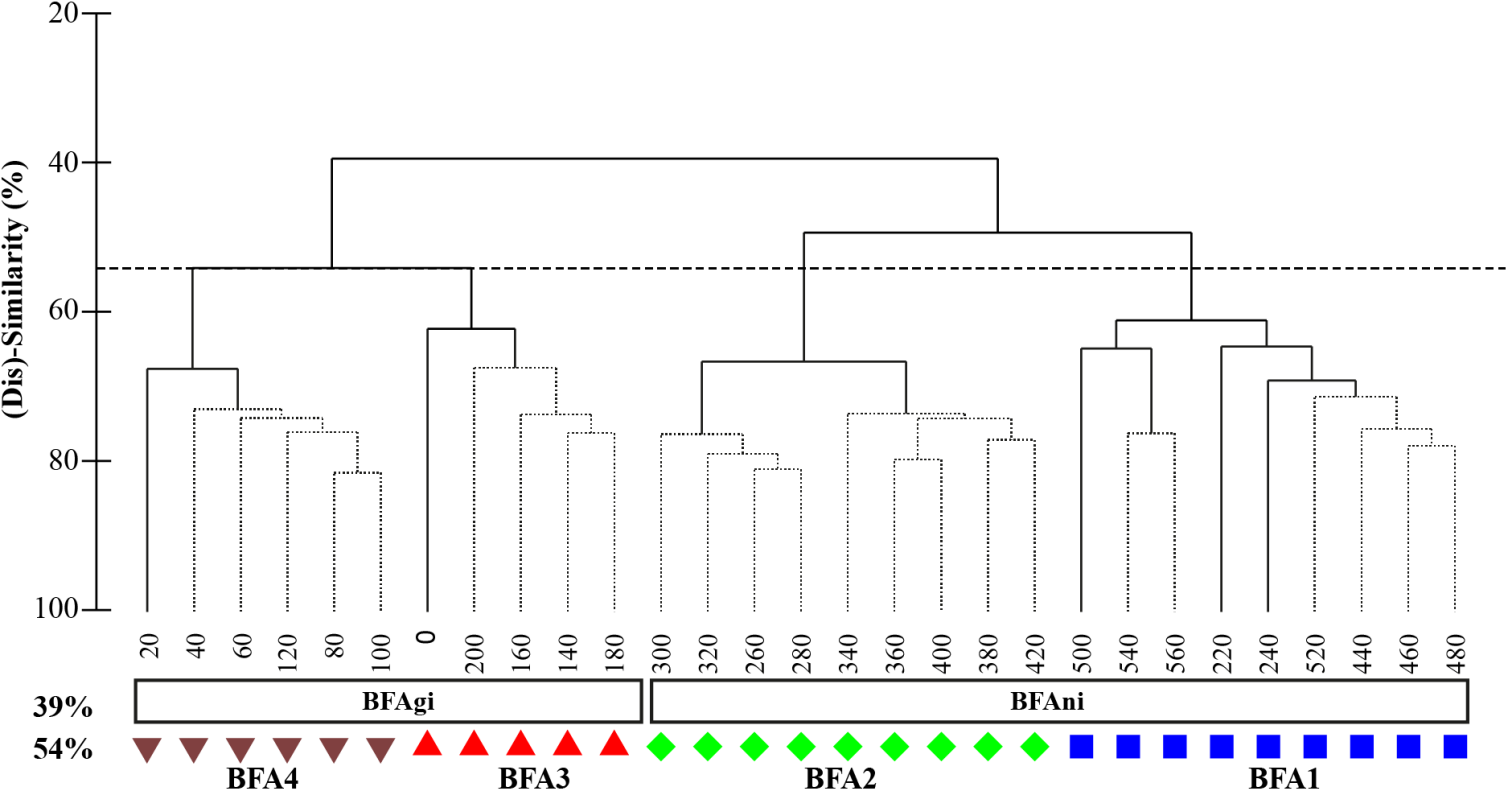
Hierarchical dendrogram based on the Bray-Curties similarity matrix of benthic foraminiferal compositional dataset from core TTR17-401G. Two clusters are separated at 39% of similarity (BFAgi and BFAni). At 54% of similarity, four clusters can be recognized (BFA1, BFA2, BFA3 and BFA4).

The hierarchical cluster analysis based on the Bray-Curties similarity matrix of benthic foraminifera compositional data of core TTR17-401G indicates that two clusters can be distinguished already at relatively low similarity level (39%) (Fig 6). The first cluster (BFAni) groups the samples 220–560 cm corresponding to the interval dominated by *N*. *incompta* whereas the second cluster (BFAgi) groups the upper samples of the core (0–200 cm) dominated by *G*. *inflata* (Fig 6). Thirty-two species account for 90.35% of the average similarity of the cluster corresponding to the *N*. *incompta* interval (S7 Table). This cluster is dominated by *C*. *laevigata* and *C*. *lobatulus*. Thirty-two species account for 90.38% of the average similarity of the cluster covering the *G*. *inflata* interval, which is dominated by *B*. *marginata* and *B*. *aculeata* (S7 Table).

By increasing the similarity level to 54%, the latter two clusters can be further subdivided into 4 clusters (Fig 6). Cluster BFA1 comprises the lowermost samples (440–560 cm) of the core and samples 220–240 cm. Thirty-one species account for 90.68% of the average similarity (S8 Table). This cluster is dominated by *C*. *lobatulus* and *C*. *laevigata* together with the major accessory species *R*. *bradyi*, *N*. *turgida* and *C*. *pachyderma*.

Cluster BFA2 contains the samples 260–420 cm (Fig 6). Thirty species account for 90.32% of the average similarity. Dominant species are *C*. *laevigata* together with the epibenthic *C*. *ungerianus*, *C*. *pachyderma*, *D*. *coronata* and *C*. *lobatulus* (S8 Table).

Samples 140–200 cm and 0 cm belong to cluster BFA3 (Fig 6). Twenty-four species contribute to 90.69% of the average similarity. Cluster BFA3 is dominated by the *B*. *marginata* and *B*. *aculeata* whereas *C*. *laevigata* together with *H*. *balthica* and *M*. *barleeanum* are major accessory species (S8 Table).

The last cluster BFA4 comprises the upper samples between 20 and 120 cm (Fig 6). Thirty-five species account for 90.88% of the average similarity and major contributors are *C*. *lobatulus*, *B*. *marginata* and *B*. *aculeata* together with *G*. *praegeri* and *C*. *ungerianus* (S8 Table).

#### Ostracods–distribution and assemblages

The ostracod distribution pattern allows a separation of the assemblages in three groups corresponding to the benthic foraminiferal assemblages BFA1, BFA2 and BFA3-4. In the intervals BFA1 and BFA3-BFA4 the abundance and diversity of ostracods is generally low (Fig 2, S3 Table). Highest abundances and species richness in ostracods are reached in the interval corresponding to BFA2 and coincides with a general decrease of the clay fraction in the interval 260–420 cm. Individual maxima (540, 420, 260 cm) coincide with elevated abundances and/or diversities of benthic macrofauna (Fig 2). The highest abundance and species richness (9 species) of ostracods at 420 cm is coupled with a significant reduction of the mud fraction and TOC.

### Benthic macrofauna–abundance and diversity

The benthic carbonate components between 1 and 10 mm in size consist, in order of abundance, of scleractinian corals, bryozoans, serpulids, bivalves, echinoderms, spirorbids, verrucids, brachiopods and secondarily foraminifera. They are not uniformly distributed along the core, but are particularly abundant in the 280–300 cm and 420 cm intervals and almost absent at 160 and 500 cm. Corals, bryozoans and serpulids represent over 60% of the macrofauna assemblage of almost all analysed samples and bryozoans are the most diverse taxonomic group along the core. Corals consist of two typical frame-building scleractinian species, *Madrepora oculata* and *Lophelia pertusa*, and secondarily of dendrophylliids whose small fragments (presumably belonging to the species *Dendrophyllia cornigera*) do not allow unequivocal identification. *Lophelia pertusa* dominates the interval 260–520 cm and its largest and best-preserved fragments have been found at 300–320 and 440–460 cm sediment intervals (Fig 2).

In the upper 240 cm of the core *M*. *oculata* is associated to dendrophylliids (Fig 2) and shows large (up to 7 cm in length) and well-preserved fragments. This species is less common in the lower part of the core and is absent between 300 and 520 cm.

The bryozoans identified along the core belong to 30 species, mostly typical of the outer shelf (some extending to deep waters), but only few of them are common in all samples. Excluding erect flexible remains of representatives of the Family Candidae and the genus Crisia, very rare along the core and restricted to the upper 200 cm, the identified bryozoans can be assembled in three main groups:

1. Cheilostomes showing erect rigid growth morphologies, consisting of essentially three species (*Buskea dichotoma*, *Palmiskenea gautieri* and *Reteporella sparteli*),
2. Erect Tubuliporina cyclostomes, largely represented by *Tervia irregularis*, and species of *Entalophoroecia*, *Idmidronea* and *Tubulipora*;
3. Encrusting morphotypes, which are overall subordinate in the core loose sediment though their etching traces are locally abundant on coral fragments.

In Fig 2 the increased abundance of the first bryozoan group is clearly shown in the lower two meters of the core where the dominant species *B*. *dichotoma* displays a rise in both abundance and fragment size. Serpulids occur throughout the core but are particularly abundant in the samples collected at 320 and 420 cm where the aggregated tiny tubes of the genus *Filograna* (both in loose sediment and still attached to coral fragments) represent over 30 and 20%, respectively, of the macrofauna assemblages.

Although most identified macrofauna taxa occur all along the core, some of them show narrower distribution ranges. In particular, ostreid bivalves and the bryozoan Candidae species occur only in the upper part of the core dominated by *M*. *oculata* and Dendrophylliidae, whereas a tiny rissoid gastropod species (*Alvania* cf. *zylensis*), the bryozoans *Idmidronea triforis*,? *Tubulipora notomale*, *Hemicyclopora multispinata*, the serpulid *Filograna* and verrucids have been found only in the lower part of the core dominated by *L*. *pertusa* (Fig 2). Moreover, excluding corals, the average diversity of the benthic assemblage in the *Lophelia*-dominated interval 260–420 cm (corresponding to BFA2) is 8 and 6% higher than in the *Madrepora*- dominated upper interval (0–260 cm) and in the lower core part (420–560 cm), respectively.

## Discussion

### CWC micro- and macrofauna related to paleoceanography and climate variability in the MMF

The interval showing a predominance of the scleractinian coral *L*. *pertusa* is characterized by the occurrence of the benthic foraminiferal assemblage BFAni dominated by *C*. *laevigata and C*. *lobatulus*. Both species are common in CWC fossil records from the Norwegian shelf [25], the Porcupine Seabight [26] and from the western Alboran Sea [31] where they are associated to nutrient rich, well-oxygenated and relatively high-energy bottom waters. In the western Mediterranean Sea, Milker and Schmiedl [68] have demonstrated that *C*. *lobatulus* was a dominant species in the latest glacial benthic foraminiferal assemblages and that it became progressively less abundant since ~6.5 ka BP in the Alboran Sea. This species represents however still an important taxon of the recent shallow shelf environments (40–80 m water depth) in the Western Mediterranean Sea and shows a strong correlation to coarse substrates such as biogenic sand and gravel [81].

Milker and Schmiedl [68] report *C*. *laevigata* from the last ~3.5 ka BP and at very low abundances around the Alboran Island whereas this species is a major contributor of the recent (living) benthic foraminiferal community in the Oran Bight and the Mallorca shelf. Milker et al. [81] noticed that *C*. *laevigata* was mainly restricted to fine-grained low energy deposits on the Mallorca Shelf whereas on the Alboran Platform and the Oran Bight it was often associated to typical high-energy taxa such as *C*. *refulgens*, *C*. *lobatulus* and *G*. *praegeri*. Therefore, Milker and Schmiedl [68] concluded that the distribution of *C*. *laevigata* is not uniquely governed by water depth, hydrodynamic energy and substrate but also by lateral advection of sediments and particulate organic matter.

Other studies (e.g., [82,83]) have demonstrated that high occurrences of the opportunistic shallow infaunal taxon *C*. *laevigata* in the Mediterranean Sea is mainly controlled by the availability of high labile organic carbon fluxes to the seafloor. Schmiedl et al. [84] argued that *C*. *laevigata* proliferated during the glacial periods in the Mediterranean Sea in relation to increased productivity triggered by a shallow nutri- and pycnocline and the subsequent formation of a deep chlorophyll maximum layer. Thus, the occurrence of BFAni clearly points towards a benthic environment characterized by intensified near-bottom currents providing the MMF with an important supply of fresh organic matter. Elevated primary production rates in the surface waters are in agreement with the predominance of the cool and eutrophic indicator *N*. *incompta* [31,85].

The presence of near bottom currents and the significant supply of fresh organic matter from the surface were possibly responsible also for the relatively higher diversification of bryozoans (mean species number of 3.05 within BFAni vs. 2.54 in the upper part of the core) and the proliferation of erect bryozoan species (especially *Buskea dichotoma*) which show larger size and account for much higher percentages of the macrofauna associated to corals (Fig 2, S5 Table). Moreover also the mean number of specimens and the relative taxonomic richness of the counted macrofauna is higher within BFAni than BFAgi (Fig 2, S6 Table).

The planktonic foraminifera faunal overturn at ~8 kyr BP marks the onset of the modern oceanographic conditions in the Alboran Sea and is accompanied by a deepening and a strengthening of the nutri- and pycnocline between Atlantic and Mediterranean waters [79]. The new oceanographic settings are set up in a context of a mean sea-level rise of about ~70–80 m since the late YD and increased inflow of Atlantic Waters into the Mediterranean Sea [86]. The synchronous shift in the planktonic and in the benthic foraminifera fauna from BFAni to BFAgi provides evidence for a large scale impact on the marine microfauna and seem to be confirmed by modifications in the benthic macrofauna of the Alboran Sea. *Madrepora oculata* becomes dominant among the CWCs and coral-associated taxa seem to disappear (Fig 2, S6 Table). The establishment of assemblages similar to BFAgi dominated by the shallow infaunal *B*. *marginata* and the deep infaunal *B*. *aculeata* is usually associated to high abundance of organic matter of eventually low nutritive value and decreased oxygen content (e.g., [87–91]). Van der Zwaan and Jorissen [92] demonstrated that the distribution of benthic foraminifera in the Adriatic Sea is closely related to the Po river plume which favours the accumulation of organic matter within a large clay belt. They postulated that in the vicinity of the river inflow, *B*. *marginata* is the most tolerant species regarding oxygen availability whereas the most tolerant species for the normal saline marine conditions is *B*. *aculeata*. Moreover, Jorissen et al. [93] showed that *B*. *marginata* can be regarded as a potentially infaunal dweller and is able to migrate to the uppermost sediment layer under temporary dysoxic conditions and thus take advantage of high food availability. Thus, we interpret assemblage BFAgi as indicative of an environment characterized by enhanced availability of organic matter on and in the sediment probably favoured by lower oxygen content at the seafloor. Compared to BFAni, the C_org_ deposited during the interval of BFAgi is clearly of lower quality. Furthermore, an increase of the mud fraction in the upper part of the core indicates a general decrease in bottom water energy from BFAni to BFAgi.

The shift from a CWC community dominated by *L*. *pertusa* to *M*. *oculata* and Dendrophylliids indicates that *L*. *pertusa* is comparatively more sensitive to changes in the food quality and/or oxygen content. A shift in coral composition has been previously noticed by Wienberg et al. [37] in the Gulf of Cadiz and Malinverno et al. [36] in the Ionian Sea. Moreover Vertino et al. [94] highlight the distribution in the Mediterranean between Pleistocene *Lophelia*-dominated CWC bioconstructions including cold stenothermic species (today extinct in this basin but still living in some locations of the NE Atlantic), and modern less diversified *Madrepora*-dominated communities.

**Fig 7:**
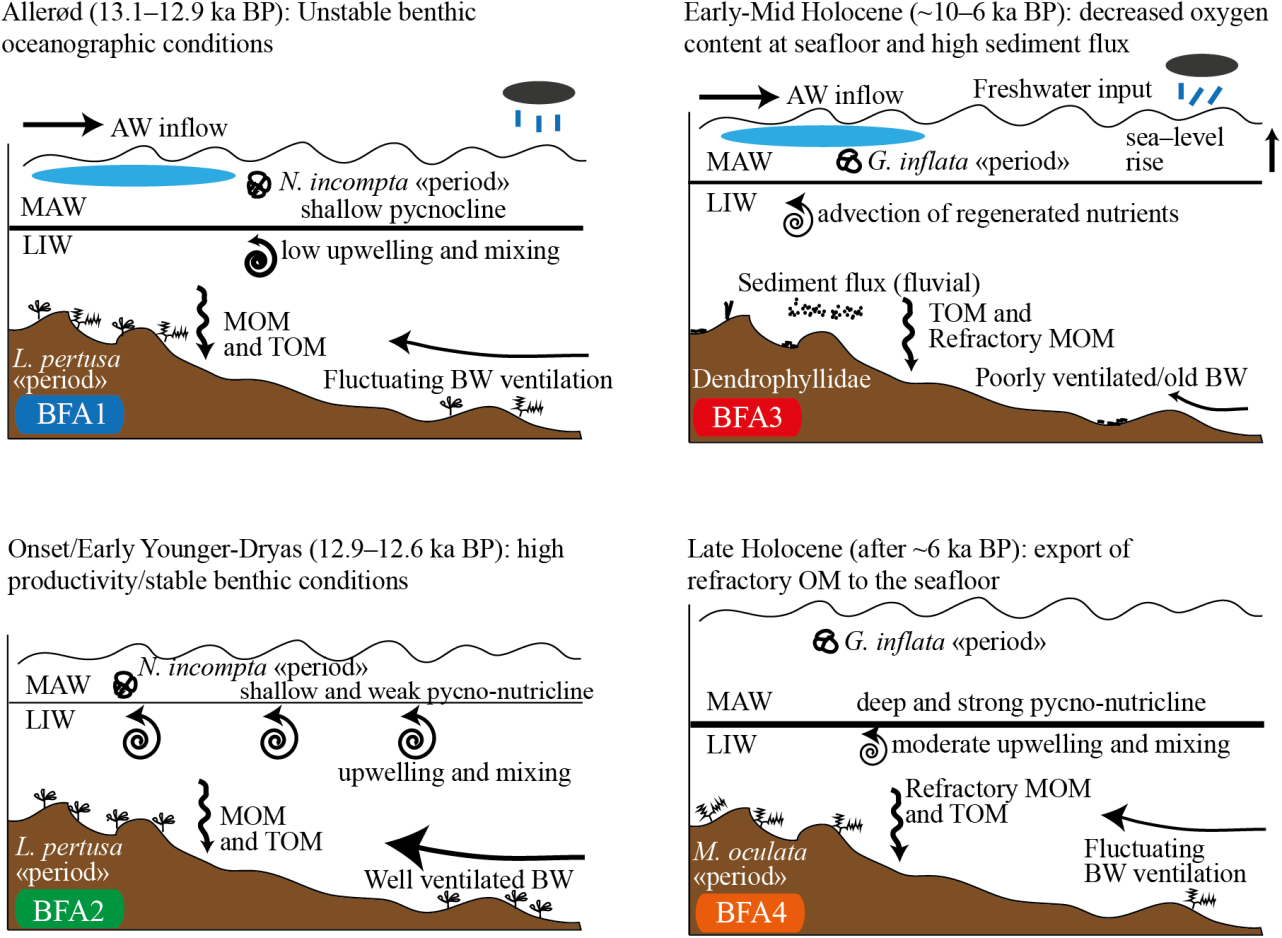
Sketch showing the evolution of cold-water corals in the MMF in the last 13 ka. The time frame and the paleoceanographic conditions are inferred from radiocarbon dating, benthic foraminiferal assemblages BFA1-4, macrofaunal data, benthic and planktonic δ^18^O and δ^13^C, Rock-Eval pyrolisis and δ^13^C_org_. Climatic and oceanographic settings included in the sketch are Atlantic Water (AW) inflow, Modified Atlantic Water (MAW), Leventine Intermediate Water (LIW), relative sea level, strength and position of the pycno-nutricline, humidity in the northern African hinterland, sediment load to the shelf, regeneration and upwelling of nutrients (e.g., phosphorus), terrigenous (TOM) and marine organic matter (MOM) and bottom water (BW) ventilation.

A close-up of our micro- and macrofossil data allows a subdivision of the periods corresponding to the assemblages BFAni and BFAgi into four distinct periods governed by different paleoclimatic and paleoceanographic conditions (Fig 7). This is well supported by the geochemical data.

The statistical subdivision of BFAni into BFA1 and BFA2 shows that BFA1 is dominated by *C*. *lobatulus* and *C*. *laevigata* together with *R*. *bradyi*, *N*. *turgida* and *C*. *pachyderma* and BFA2 by *C*. *laevigata* together with the epibenthic *C*. *ungerianus*, *C*. *pachyderma*, *D*. *coronata* and *C*. *lobatulus*. All of these species are common on CWC settings [95]. Nevertheless, the strong contribution of *N*. *turgida* and *R*. *bradyi* in BFA1 and the predominance of attached epibenthic species in BFA2 coupled with large fluctuations in the foraminiferal density reveals temporal variability in bottom water energy and oxygenation, and nutrient supply. Opportunistic taxa such as *N*. *turgida* are known to respond very fast to seasonal eutrophic blooms and are able to grow and reproduce extremely rapidly ([96] and references therein). The epibenthic *R*. *bradyi* is a common species in Mediterranean shallow shelf environments and is usually attached on elevated substrates such as e.g., *Posidonia* meadows, coarse detrital and biogenic sand and gravels [97–100]. However, Fontanier et al. [101] noticed from their experiments on living benthic foraminifera from the Bay of Fréjus yield in an incubated core that *R*. *bradyi* can be considered as an euryhaline species eventually tolerating drastic oxygen depletion. Similar observations were reported by Heinz et al. [102]. Periodically decreased oxygen concentration at the seafloor is sustained by the relatively important contribution of intermediate to deep infaunal species such as *Bolivina alata*, *B*. *difformis*, *Bolivina dilatata*, *B*. *subaerensis*, *B*. *aculeata*, *Cassidulinoides brady and Fursenkoina complanata* in BFA1 compared to BFA2 (S8 Table) (e.g., [82]).

Thus, BFA1 records relatively well-ventilated bottom waters with high C_org_ flux of labile organic matter (OM) under high primary productivity and periodically dysoxic conditions at the seafloor at the end of the Alleröd Interstadial. The signal from benthic and planktonic foraminifera is supported by the high P/B ratios, the low MINC values (Fig 4) and the fluctuating epibenthic δ^13^C and the high δ^18^O values (Fig 3). The episodic low planktonic δ^13^C and δ^18^O values during this interval suggests the occurrence of isotopically light water masses that are most likely related to a freshening of the surface waters caused either by increased Atlantic Water inflow and/or continental river runoff and enhanced precipitations over the Mediterranean Sea. The scarcity of ostracods and macrofauna in this interval highlights the rather unstable benthic oceanographic conditions (Fig 2). Absence of ostracod species during the Böllig-Alleröd Interstadials has been linked to increased freshwater influx [103].

Similar scenarios with prevailing humid and warm climatic conditions and increase of river discharge have been reported from the Alboran Sea for the Böllig–Alleröd Interstadials (e.g., [104–106]). The increase in humidity and temperature at this time is accompanied by the development of temperate forest as inferred from the pollen based climate reconstructions of the Alboran Basin [107,108]. Similarly, pollen based reconstructions of mean temperatures from west and central Europe show that temperatures were similar to present [109,110].

Several authors (e.g., [111,112]) have demonstrated that the Böllig–Alleröd is characterized by a general increase of the marine productivity whereas the oxygen concentrations of the bottom water masses are low. Decrease in the bottom-water ventilation triggered by continental freshwater discharges and meltwater inputs from the Atlantic Ocean during the deglaciation are well documented in the Alboran Sea [66,113]. According to Rogerson et al. [114], large-scale drops in the surface water density linked to ice-sheets retreat and global sea-level rise occurred in the Alboran Sea at Terminations 1a (~14.5 ka BP) and 1b (~11.6 ka BP). During these periods, the deep and epibathyal Alboran Sea, is characterized by a low density and low diversified benthic foraminiferal assemblage, which was attributed to decreased deep-water formation [65].

The establishment of assemblage BFA2 at 12.9 ka BP implies well-oxygenated benthic conditions with the occurrence of the epibenthic *D*. *coronata* and abundant *Cibicides* spp. and *Cibicidoides pachyderma* (Fig 2). *Discanomalina coronata* has been associated to high energy and well-oxygenated seafloor [27]. The strong increase of these epibenthic taxa together with *C*. *laevigata* points also to important supply of C_org_ at the water-sediment interface during the early YD. The elevated benthic foraminifera productivity with the decreasing P/B ratio and the general coarsening-up recorded during the BFA2 interval strongly support the assumption of increased bottom water circulation in the Alboran Sea. Assemblage BFA2 coincides with the highest epibenthic and planktonic δ^13^C and δ^18^O values and indicates enhanced productivity and that seafloor oxygenation increased in the MMF during this interval. Furthermore, the extremely stable epibenthic δ^13^C and δ^18^O values during this period indicate that the occurrence of assemblage BFA2 is likely linked to stable benthic oceanographic conditions, also conducive of high abundance and diversity of macrofauna and ostracods (Fig 2). We suggest that during the onset of the YD and at least until 12.6 ka BP, the MMF experienced lateral advection of nutrient and oxygen rich LIW that was accompanied by a shoaling of the nutricline. Similar models (e.g., [115,116]) have been described in the eastern Mediterranean Sea with formation of a deep chlorophyll maximum promoted by the shoaling of the nutricline within the euphotic layer. Comparable increases of ostracod abundance and species richness as well as an assemblage change have been reported in the YD of the Mediterranean. Angue Minto'o et al. [117] report a shift from a *Paracypris polita* dominated assemblage during the last glacial period to a diverse assemblage in the YD with higher percentages of *Krithe* spp. and Macrocyprididae comparable to the development in BFA2.

Ecological preferences of individual Recent ostracod species or genera are scarce or even contradictory. For instance, the tolerance to dysoxic conditions of the genus *Cytherella* sensu Whatley (1991) was extensively discussed in Dingle [118].There is however a general consensus that ostracods do not tolerate truly anoxic conditions, though there are assemblages seemingly adapted to somewhat reduced levels of bottom oxygenation [118]. This coincides with the observation of increased bottom water ventilation and higher species richness and abundances during BFA2. Since the genus *Krithe* has been attributed to the endobenthos [119–121], its relatively strong presence in BFA2 also hints at an oxygenation of the uppermost sediment layer. Occurrence peaks of the genus *Echinocythereis* in so-called “cold-assemblages” has been linked to a mixed water column and abundance of nutrients [122]. During the YD, peaks of allochthonus ostracods have been reported from the Mediterranean [117,123]. The only species from our core that may belong to the latter is *Lanceostoma lanceolata*, a species of the predominantly phytal family Paradoxostomatidae, which is generally recorded from rather shallow depths [124,125].

**Fig 8:**
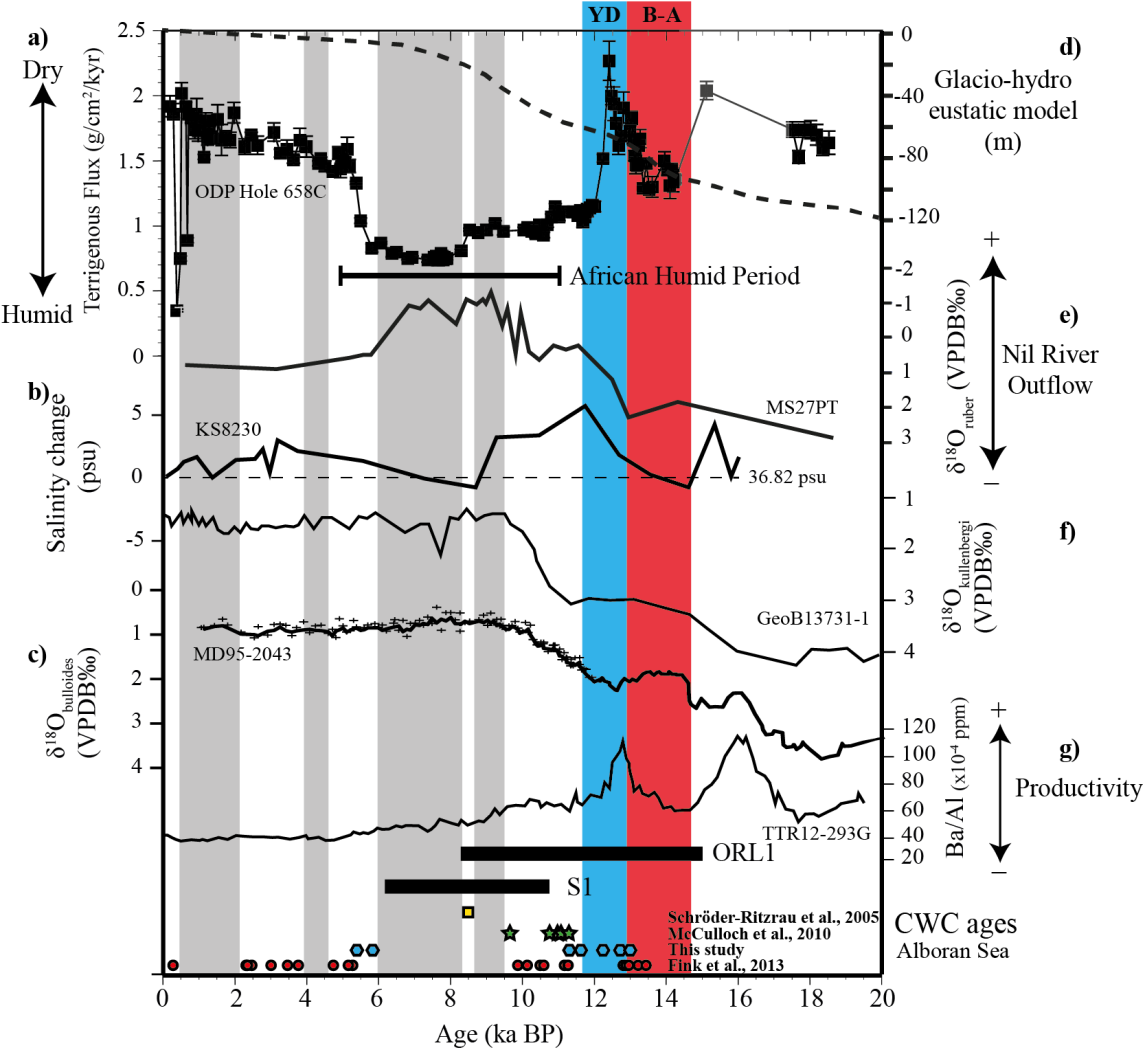
Multi-proxy record versus age. Including: a) excess ^230^Th normalized fluxes of terrigenous material at ODP Hole 658C [128], b) salinity reconstruction at KS8230 [129], c) planktonic δ^18^O (*Globigerina bulloides*) at MD95-2043 [114], d) glacio-hydro eustatic model for the western Mediterranean Sea [86], e) planktonic δ^18^O (*Globigerinoides ruber* var. alba) at MS27PT [130], f) benthic δ^18^O (*Cibicides kullenbergi*) at GeoB13731-1 [42], g) Al-normalized concentrations of Ba (Ba/Al) at TTR12-293G [106]. At the lower panel are displayed the ages of corals dated in the Alboran Sea. The grey dashed areas indicate possible periods of interruption of CWC growth during the last 14 ka in the Alboran Sea.

High fertile and productive surface waters during the YD in the eastern Alboran Sea have been documented from several sediment records based on Ba/Al ratio [106,126] (Fig 8), diatoms [104] and coccolitophores [112,127]. According to Fink et al. [42], enhanced marine productivity occurs at the beginning of the YD in the MMF. Increased productivity at the onset of the YD is also corroborated by significant increase of Barium excess (i.e., marine Barite) in three sediment cores recovered in the easternmost Alboran Basin [111]. These authors postulated that episodic torrential rainfalls accompanied by massive river discharge during the generally cold and arid YD promoted nutrient and sediment transport to the Alboran Basin.

Caralp [65] showed that benthic foraminifera became more abundant and more diversified during the YD compared to the Böllig–Alleröd with increasing abundances of the epibenthic *Cibicides pseudoungerianus* in the epibathyal part of the Alboran Sea. Based on a synchronous increase of benthic foraminifera diversity and abundance on the eastern side of the Gibraltar Strait, Caralp [65] concluded that the east-west flow was enhanced during the YD. Enhanced MOW circulation through the Gibraltar during the YD has been largely described based on the occurrence of coarse-grained contourites in the Gulf of Cadiz [131–133] and near-bottom current sensitive epibenthic foraminifera [134].

The short re-occurrence of BFA1 coincides with the onset of the Holocene and the beginning of the monsoon driven African Humid Period (AHP; ~11–5 ka BP) with increased meltwater inputs from the vanishing central and western European ice-caps [134, 135]. A shift towards humid conditions on the northern African continent is also inferred from marine records with an abrupt drop of wind blown dust at ODP site 658C off Cape Blanc [128] and depleted δ^18^O of the planktonic surface dweller *Globigerinoides ruber* in the eastern Mediterranean Sea due to increased outflow of the Nil river [130] (Fig 8). The end of the YD is marked by a drastic warming of more than 5°C in less than 200 years and by strong fluctuations in the sea-surface temperature (SST) in the western Mediterranean Sea [111]. Our planktonic δ^18^O follow a similar trend and similar values as observed in the planktonic δ^18^O (*G*. *bulloides*) from adjacent areas [114] (Fig 8).

The establishment of a significantly distinct benthic foraminiferal assemblage (BFA3) concomitant with a planktonic foraminiferal fauna dominated by *G*. *inflata* indicates that a major part of the early Holocene is not preserved in our record (see below). This assemblage is dominated by *B*. *marginata* and *B*. *aculeata* but shows a strong contribution of *C*. *laevigata* together with *H*. *balthica* and *Melonis barleeanum*. Both the epifaunal *H*. *balthica* and the infaunal *M*. *barleeanum* are typical taxa found on shallow shelf down to deep bathyal environments [136]. In the Mediterranean Sea, *H*. *balthica* is more abundant at water depths between 400 and 500 m where temperatures vary from 14.5 to 15°C and salinity from 38 to 39 [137–139]. In the northern Atlantic Ocean, *H*. *balthica* thrives usually in cold waters with temperatures ranging from 4 to 7.5°C [140,141]. It has been widely used as an indicator for cold shelf water masses (e.g., [142,143]) and seems to proliferate in the Alboran Sea during cold climatic episodes such as the YD [65].

The distribution of *H*. *balthica* in the Mediterranean Sea has been reported from high-diversified benthic foraminiferal assemblages and associated to favorable benthic conditions [89,144]. However, Drinia et al. [145] found a benthic foraminiferal assemblage in the central Aegean Sea dominated by *B*. *marginata*, *C*. *laevigata* and *H*. *balthica* similar to BFA3 and inferred that this assemblage is indicative of an unstable benthic environment with seasonal pulses of C_org_. Similarly, Fontanier et al. [101] noticed that *H*. *balthica* was dominant in the thanatocoenoses of the Bay of Fréjus and suggested that this species has an opportunistic behavior with regard to C_org_ enrichment at the seafloor. This is well in agreement with distribution of *H*. *balthica* in the eastern Mediterranean Sea where this species becomes more abundant as the C_org_ fluxes start to increase and the bottom water oxygen content drop shortly before the deposition of sapropel S5 [146]. Similar distribution patterns are observed for *M*. *barleeanum* at the base of sapropel S6 and at lower extend at the base of S5 [146]. This intermediate infaunal species is known to occur in meso- to eutrophic environments and is able to withstand periodically dysoxic to anoxic conditions (e.g., [88,147]). Furthermore, Fontanier et al. [90] argued that it tolerates low quality organic matter and may feed on bacteria. Thus, we interpreted assemblage BFA3 as indicative of an environment with C_org_-enriched sediments favored by decreased oxygen concentrations at the seafloor and periodically strong seasonal plankton blooms (Fig 7). The occurrence of oxygen-depleted bottom waters at this time is corroborated by the remarkably low benthic δ^13^C values, the low benthic foraminifera diversity and the highest contribution of typical low oxygen indicators such as *Cassidulinoides* spp., *Chilostomella* spp., *Fursenkoina* spp., *Globobulimina* spp. The lowest MINC values and the elevated P/B ratio testify that the benthos suffered oxygen depletion. Moreover, the increase of the mud fraction during this interval corroborates low bottom water circulation in the MMF. The extent of this comparatively stressed benthic environment is also visible in the ostracod community and the macrofauna with a drastic drop in diversity and abundance. The restriction of Dendrophylliids in interval BFA3 can be tentatively interpreted as the proliferation of opportunistic genera with a relatively high tolerance to stressed environments.

The gradual increase in the benthic δ^18^O during this interval most likely reflects the presence of cold and/or dense bottom water masses, which may be explained by advection of deeper and/or older water masses. If so, the reduced vertical water density contrast possibly promoted injection of regenerated nutrients and isotopically light carbon (^12^C) into the upper water masses as suggested by the strongly depleted planktonic foraminiferal δ^13^C [111,148]. The physical shoaling of intermediate water (LIW) together with a decrease of surface water salinity is a possible trigger mechanism for the deposition of the C_org_-enriched sediments (ORL1) in the Alboran Sea [114]. Furthermore, this time span corresponds to the deposition of the last sapropel in the eastern Mediterranean Sea and is accompanied by a drastic decrease of deep-water formation and reduction of the thermohaline circulation in the Mediterranean Sea [149,150].

Since interval BFA3 coincides with the timing of a major oceanographic change in the Alboran Sea we assume that assemblage BFA3 reflects the rearrangement of the water mass configuration and the rapid flushing of old deep water after the perturbation of the thermohaline circulation in the Mediterranean Sea during sapropel S1a. Thus, the gradual increase in the planktonic δ^18^O mimics the progressive increase in salinity of the surface waters, which is well in agreement with the salinity reconstruction of the Alboran surface waters proposed by Emeis et al. [129].

Moreover, the hydrographic settings and the trophic structure of sub-surface waters of the Alboran Sea are linked to density contrasts with the inflowing Atlantic Water and to the variability in the activity of the two anticyclonic gyres, becoming extremely complex at shallower water depths in relation to topographically induced local upwelling zones. The MMF is composed of several hundreds of small to large size mounds shaped by strong bottom currents as indicated by the occurrence of moats at their base, visible on seismic profiles [45]. Even short-term variability in the position (and strength) of the EAG and in the hydrographic circulation together with climate forcing would most likely impact the bio-geochemical settings of both the water column and the seafloor.

The shift in the benthic foraminifera community from BFA3 to BFA4 coincides with an increase in benthic foraminiferal diversity and corresponds to a strong increase of *C*. *lobatulus* which become dominant together with *B*. *marginata* and *B*. *aculeata* and associated fauna *C*. *laevigata* and *G*. *praegeri*. The strong contribution of the epibenthic species *C*. *lobatulus* and *G*. *praegeri* and significant abundances of *C*. *ungerianus* and *C*. *pachyderma* indicate that the benthic environment at the MMF was characterized by a return to more oxygenated conditions during this interval. This is well corroborated by the sharp increase of the benthic δ^13^C values and the higher MINC values. According to the benthic δ^18^O values together with a strong decrease of *H*. *balthica*, the bottom waters of the MMF experienced during this time a warming and/or became less dense compared to BFA3. Relatively high planktonic δ^18^O values together with dominance of *G*. *inflata* in the plantktonic foraminifera community are well in agreement with a deeper and well established pycno-nutricline at this time.

The establishment of BFA4 is accompanied by a more diversified and abundant macrofauna, especially with the continuous occurrence of marine bivalves belonging to the Ostreidae family. The CWC community is almost exclusively composed of *M*. *oculata*.

However, the overall strong contribution of buliminiids and the episodic significant presence of opportunistic taxa such as *N*. *turgida*, *R*. *bradyi* together with low oxygen tolerant species such as *Chilostomella oolina* provide evidence for still variable benthic oceanographic conditions but with a relatively stable water column. This is well verified in the fluctuating benthic and planktonic isotopes and is in good agreement with previous data reported after deposition of ORL1 in the eastern Alboran Sea [111].

The short re-occurrence of BFA3 in the uppermost part of core TTR17-401G marks a return to less favorable environmental conditions for the benthic fauna with the presence of dense and oxygen depleted water mass at the seafloor (Fig 7). Our micro and macrofaunal data together with the geochemical proxy provide evidence for relatively important fluctuations of the oceanographic conditions at the MMF during the Holocene. This confirms the occurrence of several short SST cooling of the Alboran Sea induced by inflowing AW and sustained cold continental winds reported by Cacho et al. [151]. Furthermore, climate variability during the Holocene in the Western Mediterranean region is also well recognized in changes of vegetation with episodes of forest decline due to dry atmospheric conditions [108].

We suggest that such bio-geochemical and physical variability in the benthic boundary layer, most probably intensified at shallower water depth, may likely be responsible for CWC decline in the MMF.

### Presence of hiatuses

Declining or buried CWC structures are weakened through bioerosion and physico-chemical alteration and thus are better exposed to erosion [152]. Our data provide evidence for periodically reduced or absent CWC growths in the MMF and strong erosion by currents in two, maybe four horizons along the core (Fig 3). Hiatuses may be characterized by age differences between corals and sediments, by the presence of coarser sediments [153], of poorly preserved corals or negative δ^13^C excursions [18], excursions to higher carbonate content [19] or abrupt faunal changes [26]. The two horizons at ~250 and ~430 cm depth are characterized by negative excursions in the TOC content and in the grain size distribution and coincide with major changes in the foraminifera and ostracod fauna (Figs 2 and 3). Moreover, the preservation of the CWC fragments occurring in those horizons is generally poor due to extensive bioerosion and chemical dissolution suggesting prolonged exposure at the water-sediment interface (Fig 2).

The upper two horizons at the core top and at ~200 cm depth have been identified by different ages between corals and sediments (see chapter Chronology). The discrepancy of the ages at the planktonic foraminifera turnover (~8 ka) implies that the Early Holocene is missing in core TTR17-401G. This is corroborated by the planktonic and benthic δ^18^O values dropping from typical deglacial to interglacial values. The cooling and/or increase in density of the benthic δ^18^O values of interval BFA3 correspond to similar values and trends observed in the epibenthic δ^18^O values of *Cibicides kullenbergi* (core GeoB 13731–1, 362 m water depth) from Fink et al. [42] at around 7.2–6.2 ka BP. This period succeeds a centennial (8.5–8 ka BP) cooling event characterized in the marine record of the Mediterranean Sea by high Saharan dust content and interruption of the deposition of sapropel S1 due to increased bottom water ventilation [154–156]. This period also coincides with an increase of the surface water salinity in the Alboran Sea [129] well depicted in our planktonic δ^18^O values.

### CWC mound growth rate in the MMF

**Fig 9:**
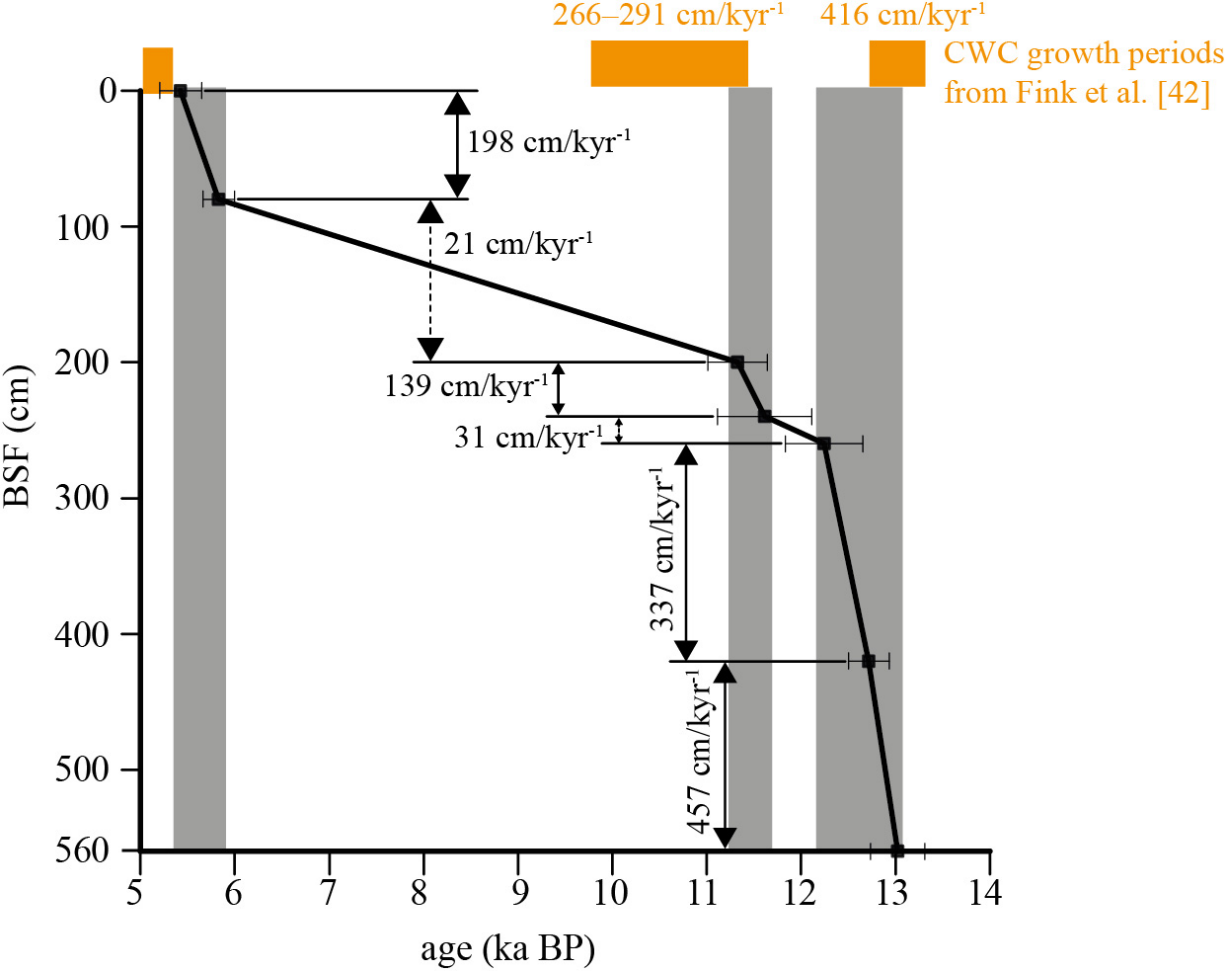
Depth versus age plot for cold-water coral samples from core TTR17-401G. Displayed are vertical mound aggradation rates. Dashed areas (grey) indicate sustained CWC growth at the Melilla Mounds Field (MMF). The panels (purple) show cold-water coral growth periods in the MMF based on radiocarbon ages of cold-water corals from Fink et al. [42] and their respective vertical mound aggradation rates.

Our faunal data indicate that CWC growth was sustained in the MMF during the late Alleröd–YD (Fig 2). The mound aggradation rate for core TTR17-401G is up to 457 cm ka^-1^ (Fig 9), which is high compared with other data from the same area [42] but rather low in comparison to the northern Norwegian shelf where aggradation rates of up to 1500 cm ka^-1^ have been reported [157]. Vertical CWC mound growth is enhanced by the sediment baffling capacity of the coral branches. In core TTR17-401G the time interval from the coral age and that of surrounding sediments (from foraminifera) does in fact not exceed 500 years and thus confirms the active sediment retention by the coral framework. Our data suggest that CWC growth in the MMF was reduced or even ceased during the late YD (31 cm ka^-1^) and shortly after the early Holocene and lasted at maximum until 5.8 ka BP (21 cm ka^-1^) (Fig 9). This agrees well with the coral ages reported by Fink et al. [42] showing that CWC occurred in the MMF from 13.5–12.8, 11.4–9.8, 5.4–2.3 and at 0.3 ka BP. All changes in the aggradation rates are preceded by the establishment of different benthic foraminiferal assemblages highlighting that benthic foraminifera can be used as reliable proxy for paleoceanographic reconstruction and also for estimate the evolution through time of certain macrofauna such as CWC with regard to climate and oceanographic variability.

### Organic matter–sources, fluxes and preservation

How much climate variability and the distance to the North African continent affected the ecosystems of the MMF is also highlighted by the provenance of the C_org_ exported to the seafloor. The pseudo-Van Krevelen graph [80] (Fig 5) shows that all samples plot at the boundary of the type II kerogen field (marine) and III (terrestrial) pointing towards a mix of marine organic matter (MOM) and terrigenous organic matter (TOM) [80,158,159]. The relatively low HI values and high OI is typical for (i) high input of TOM, (ii) important contribution of partially reworked continental organic matter, and/or (iii) strong oxidation of indigenous MOM.

However, two different groups can be recognized, one composed by the samples belonging to BFA1-BFA2 (BFAni) and another by samples BFA3-BFA4 (BFAgi). A more detailed definition of the source of the C_org_ is displayed in the S2 versus TOC diagram (Fig 5) where the regression lines of both samples BFA1-BFA2 and BFA3-BFA4 plot in the type III kerogen field and show a positive x-intercept indicating the presence of a matrix effect due to presence of clay minerals [80,160]. The regression line of samples BFA1-BFA2 has a slope close to the type II-III boundary suggesting a mixture of TOM and MOM, which is in good agreement with a constant value of HI of 161 [78]. The high correlation degree (r = 0.88) among these samples indicates a common origin of the C_org_ deposited in this interval (Fig 5).

The regression line of samples BFA3-BFA4 suggests a mixture of highly degraded MOM together with a relatively important contribution of TOM as indicated by the low constant HI value of 99. The source of C_org_ seems to be common to all of these samples (r = 0.85). The shift in the source of C_org_ is well corroborated by the δ^13^C_org_, which displays values typical for marine phytoplankton in the interval BFA1-BFA2 and a higher admixture of TOM in the upper part of the core, in particular during the interval BFA3. Moreover, the dominance of *B*. *marginata* and *B*. *aculeata* during interval BFAgi provides evidence for an adaptation of the benthic foraminifera to more refractory C_org_ fluxes at the seafloor after the establishment of the modern oceanographic conditions.

Increased TOM input can be linked to the AHP, whereas highly degraded MOM is most likely related to the modern oceanographic configuration. A deepening of the pycnocline associated with a higher density contrast and higher sea-level would have increased the residence time of the OM in the water column, thus leading to extensive degradation. An additional explanation for preferential accumulation of more refractory components in the sediments during interval BFA3-BFA4 are the lower mean sedimentation rates increasing the exposure time of the settling OM to oxygen fuelled bottom water masses.

The shift in the quality of the C_org_ fluxes to the seafloor from BFA1-BFA2 to BFA3-BFA4 represents a major factor that influenced the evolution of the benthos, as indicated by the faunal shift in the benthic micro- and macrofaunal community, where more tolerant species to fast changing and stressed environments are favored, e.g., *M*. *oculata* and Dendrophylliidae in the CWC community (Fig 7).

### CWC development in the Alboran Sea during late Pleistocene/Holocene

Information on CWC evolution in the Alboran Sea since late Pleistocene is still limited, and radiocarbon and U/Th dates on CWC (Figs 1 and 8) are restricted to surface samples and gravity cores from the MMF [42], surface samples from the northeastern Alboran Sea [44] and one surface sample from the Cabliers Bank [43]. They indicate that CWC were established in the Alboran Sea at least from 13.5 to 12.8, 11.4 to 8.5, 5.4 to 2.3, and at 0.3 ka BP. Our CWC dates fit generally well within this time window although they provide further evidence for CWC occurrence during the YD and from 5.8 ka BP on. All together, these ages strongly suggest that CWC were prolific in the Alboran Sea at the transition Pleistocene–Holocene and absent between 8.5 and 5.8 ka BP and between 2.3 and 0.3 ka BP (Fig 8). As suggest by McCulloch et al. [44] the combination of different factors such as drastic increase in SST's, sediment smothering, decrease in dissolved phosphorus fluxes and changes in the potential water density through sea level and salinity control could have contributed to the demise of CWC during the Holocene in the Mediterranean Sea. Our data suggest that the most favorable environmental conditions for the micro- and macrofauna were reached in the MMF during the early YD with, compared to the rest of the record, enhanced C_org_ fluxes, cool/dense and well oxygenated bottom waters and relatively strong bottom currents. Moreover, this interval is characterized by extremely stable geochemical water conditions at the seafloor. However, CWC growth occurred in the MMF also after but at lower rates and implied possibly a change in the CWC community with increased abundances of more tolerant taxa like *Dendrophyllia* spp. and *M*. *oculata*. This needs however to be verified in other records from the Alboran Sea. Our benthic foraminifera and geochemical data indicate that the benthic boundary layer at the MMF was rather unstable during the Holocene with fluctuating oxygen concentrations, water density, sediment and C_org_ fluxes. The occurrence of several hiatuses in our sediment record reflects well the benthic environmental variability at the seafloor in the MMF with the most prominent of these events taking place around the early-mid Holocene. Cold-water coral decline during this period is most likely related to reduced thermohaline circulation in the Alboran Sea resulting at the MMF in severe oxygen depletion at the seafloor, sediment flow and fluctuating bottom water density. Return to more favorable environmental conditions occurred after the establishment of a modern hydrographic configuration, with CWC growth starting with pioneering taxa (*Dendrophyllia* spp.) presumably as soon as 7.2 ka BP.

Nevertheless, it has also to be kept in mind that the Alboran Sea is a very particular marine setting influenced both by Atlantic and Mediterranean Waters and thus CWC development can not been generalized over the entire basin on such short time scales (centennial to millennial) with regard to the important regional differences in the hydrographic, bathymetric and trophic configuration. This agrees with the findings of Margreth et al. [27] reporting the presence of a patchy CWC reef on top of a mud volcano in the western Alboran Sea from 15.6–7.6 ka BP and a well-established reef from 4.2–2.2 ka BP.

## Conclusions

The detailed and integrated study of micro- and macrofossils, coupled together with geochemical proxies, allows the following conclusions regarding the evolution of a cold-water coral mound in the eastern Alboran Sea in relation to oceanographic and climatic variability during the last 13.1 ka:

1. The late Alleröd-early Holocene is characterized by enhanced fluxes of labile organic matter whereas the mid-late Holocene (corresponding to the modern-like oceanographic configuration) is characterized by more refractory and variable C_org_ fluxes to the seafloor. This transition is accompanied by a shift from *Lophelia pertusa* to a *Madrepora oculata*-Dendrophylliidae dominated CWC community. Our data suggest that Dendrophylliidae belong to the most stress tolerant species with regard to oxygen and food availability.
2. Radiocarbon dating on cold-water corals and sediments indicate that mound aggradation rates were enhanced during the late Alleröd-early YD and became progressively reduced or may have stopped during the early-mid Holocene.
3. Benthic foraminifera assemblages dominated by epibenthic dwellers together with a diversified ostracod assemblage provide evidence for periods of stable benthic conditions with cold/dense and well oxygenated bottom waters, high fluxes of labile organic matter and relatively strong bottom currents favoring enhanced CWC growth and conducive of a high diversified and abundant associated macrofauna at the onset of the YD in the eastern Alboran Sea.
4. Large hiatuses occurring at the onset of the Holocene and the establishment of more tolerant benthic foraminifera assemblages dominated by buliminiids indicate that the MMF experienced periodically decreased bottom water energy and oxygenation, variable bottom water temperatures/densities and increased sediment flow.
5. The relatively shallow site of the MMF is closely linked to the surface water masses and to atmospheric and climatic variability. Its proximity with the African continent influences the C_org_ export, the sediment budget and water mass salinities.

Taken as a whole, our high-resolution faunal and geochemical data also highlight that the use of benthic foraminifera assemblages is crucial in order to decipher the paleorecord from complex settings such as CWC ecosystems, where sedimentation rates are highly variable and hiatuses frequent.

## Supporting Information

S1 Table Range chart of benthic foraminifera of core TTR17-401G. (XLSX)

S2 Table Range chart of planktonic foraminifera of core TTR17-401G. (XLSX)

S3 Table Range chart of ostracods of core TTR17-401G. (XLSX)

S4 Table Range chart of scleractinian cold-water corals of core TTR17-401G. (XLSX)

S5 Table Range chart of bryozoans of core TTR17-401G. (XLSX)

S6 Table Range chart of benthic macrofaunal components of core TTR17-401G. (XLSX)

S7 Table List of benthic foraminifera and statistical parameters associated with the similarity in clusters BFAgi and BFAni. (XLSX)

S8 Table List of benthic foraminifera and statistical parameters associated with the similarity in clusters BFA1-4. (XLSX)
